# The [2Fe‐2S] cluster of mitochondrial outer membrane protein mitoNEET has an O_2_ ‐regulated nitric oxide access tunnel

**DOI:** 10.1002/1873-3468.15097

**Published:** 2025-01-05

**Authors:** Thao Nghi Hoang, Meritxell Wu‐Lu, Alberto Collauto, Peter‐Leon Hagedoorn, Madalina Alexandru, Maike Henschel, Shahram Kordasti, Maria Andrea Mroginski, Maxie M. Roessler, Kourosh H. Ebrahimi

**Affiliations:** ^1^ Institute of Pharmaceutical Science King's College London UK; ^2^ Department of Pharmacy Da Nang University of Medical Technology and Pharmacy Vietnam; ^3^ Department of Chemistry Technical University of Berlin Germany; ^4^ Department of Chemistry and Centre for Pulse EPR Spectroscopy (PEPR) Imperial College London UK; ^5^ Department of Biotechnology Delft University of Technology TU Delft The Netherlands; ^6^ Comprehensive Cancer Center King's College London UK

**Keywords:** gasotransmitter, H_2_S, hypoxia, iron–sulphur cluster, mitoNEET, nitric oxide

## Abstract

The mitochondrial outer membrane iron–sulphur ([Fe‐S]) protein mitoNEET has been extensively studied as a target of the anti‐inflammatory and type‐2 diabetes drug pioglitazone and as a protein affecting mitochondrial respiratory rate. Despite these extensive past studies, its molecular function has yet to be discovered. Here, we applied an interdisciplinary approach and discovered an explicit nitric oxide (NO) access site to the mitoNEET [2Fe‐2S] cluster. We found that O_2_ and pioglitazone block NO access to the cluster, suggesting a molecular function for the mitoNEET [2Fe‐2S] cluster in mitochondrial signal transduction. Our discovery hints at a new pathway *via* which mitochondria can sense hypoxia through O_2_ protection of the mitoNEET [2Fe‐2S] cluster, a new paradigm in understanding the importance of [Fe‐S] clusters for gasotransmitter signal transduction in eukaryotes.

## Graphical abstract

The mitochondrial outer membrane iron–sulphur ([Fe‐S]) protein mitoNEET is a target of the type‐2 diabetes drug pioglitazone. Its unknown molecular function is linked to respiratory complex activity and mitochondrial function. We discovered that O_2_ protects the mitoNEET [2Fe‐2S] cluster against NO oxidation and desensitization towards reduction by H_2_S. Our discovery suggests a molecular function of mitoNEET in sensing hypoxia by mitochondria.

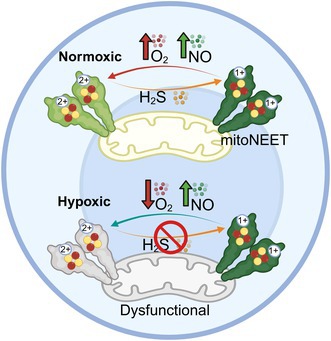

## Abbreviations

**ddhCTP**, 3′‐Deoxy‐3′,4′‐didehydro‐CTP

**EPR**, electron paramagnetic resonance

**HYSCORE**, hyperfine sublevel correlation

**MeCN**, acetonitrile

**SAM**, S‐Adenosylmethionine

**SAND**, radical SAM‐dependent nucleotide dehydratase

**TZD**, thiazolidinedione

**VDAC**, voltage‐dependent anion channel

**Viperin**, virus inhibitory protein, endoplasmic reticulum associated, interferon inducible

**Fig. 1:**
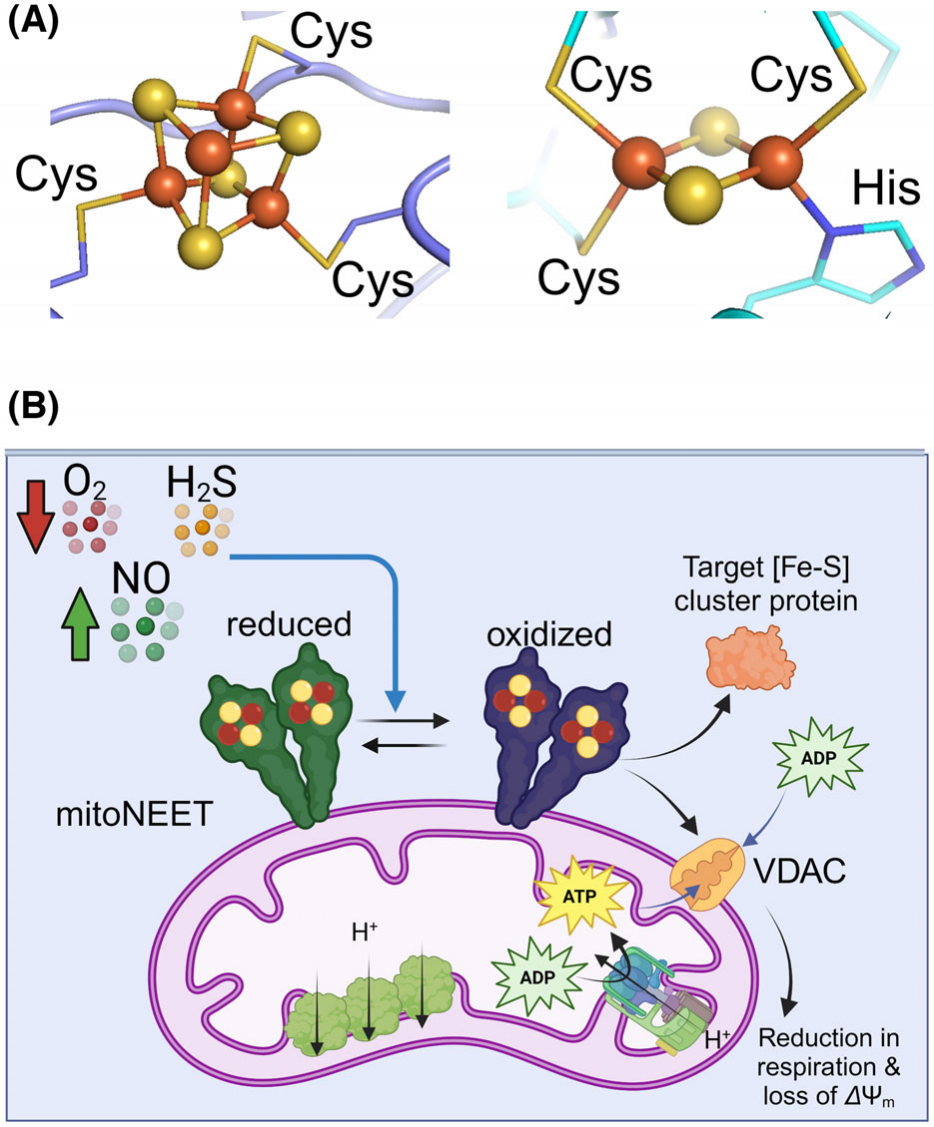
The molecular mechanism of mitoNEET function is unknown. (A) The [4Fe‐4S](Cys)_3_ cluster of radical‐SAM enzymes and the [2Fe‐2S](Cys)_3_(His) cluster of NEET family of proteins. (B) It is unknown if mitoNEET plays a specific role in sensing the levels of gasotransmitters NO and H_2_S, as well as O_2_ (blue arrow), and how this sensory function works. The expression of mitoNEET is associated with respiratory rate and mitochondrial function. Oxidized mitoNEET interacts with voltage‐dependent anion channels, thereby reducing respiration rate and causing loss of mitochondrial membrane potential (ΔΨ_m_). It is also shown that mitoNEET can transfer its cluster to the apo‐client proteins.

Proteins containing bioinorganic iron–sulphur ([Fe‐S]) clusters constitute a large structurally and genetically diverse family and have functioned in many cellular processes since the emergence of life on Earth [1]. The [Fe‐S] clusters in these proteins are synthesized using a complex machinery consisting of mitochondrial and cytoplasmic components in eukaryotes [2, 3]. This biogenesis machinery has its roots in early life before earth's oxygenation [4], highlighting the importance of [Fe‐S] clusters and proteins for all life forms. The clusters can have different stoichiometry of iron and sulphur and deviate in multiple ways from the classical all‐cysteine pattern [5, 6]. They play various functions [1]: electron transfer [7, 8], catalysis [9], sulphur donor [10], sensors of reactive oxygen or nitrogen species [11, 12, 13] and structural stability [14]. In humans, two types of clusters, namely, [4Fe‐4S](Cys)_3_ and [2Fe‐2S](Cys)_3_(His) (Fig. 1A), are found in several [Fe‐S] proteins playing vital roles in the immune response and mitochondrial function [1, 15]. The [4Fe‐4S](Cys)_3_ cluster is present in the radical S‐adenosylmethionine (SAM) enzymes [16]. These proteins have various cellular functions in humans [17]. The activity of some radical SAM enzymes such as the human SAND (hSAND) (radical SAM‐dependent nucleotide dehydratase) [18, 19] (also known as RSAD2, or viperin in humans) and Elp3 (the catalytic subunit of elongator complex) is associated with the innate immune response function [1] or oncogene expression and oncometabolism [20]. The [2Fe‐2S](Cys)_3_(His) cluster, on the other hand, is found in members of NEET (Asn‐Glu‐Glu‐Thr) family of proteins, encoded by three genes CISD1 (encoding mitoNEET), CISD2 and CISD3 [21]. Among the proteins encoded by these genes, mitoNEET is localized at the mitochondrial outer membrane and is a target of the anti‐inflammatory and type‐2 diabetes drug pioglitazone (a class of thiazolidinedione (TZD) compounds) [22]. This protein regulates mitochondrial function and respiratory rate [23, 24]. MitoNEET expression is associated with mitochondrial dysfunction in pathophysiological conditions like cancer, diabetes, age‐related heart failure and neurodegeneration, highlighting mitoNEET as an effective drug target [22, 25, 26, 27, 28, 29, 30]. Pioglitazone targets mitoNEET to attenuate mitochondrial oxidative damage [22]. Additionally, the oxidized mitoNEET can transfer the [2Fe‐2S] cluster to an apo‐client protein, playing a role in the repair of target cytosolic [Fe‐S] proteins [21, 31, 32]. The mitoNEET [2Fe‐2S] cluster is shown to be pH sensitive [33, 34] and react with O_2_, H_2_O_2_ or nitric oxide (NO) [31, 32, 33, 34, 35, 36, 37, 38, 39, 40]. Despite these extensive past studies and the established link between mitoNEET and mitochondrial activity and respiratory rate, the molecular mechanism of mitoNEET function is not fully understood. It is unknown how NO, O_2_ or H_2_O_2_ access the cluster and react with it. We proposed that a redox imbalance changes the equilibrium between the reduced and oxidized mitoNEET, thereby altering mitochondrial respiration rate and activity (Fig. 1B) [20]. The redox imbalance in the cell could be associated with a decrease in O_2_ level (hypoxia), an increase in NO production and a change in hydrogen sulphide (H_2_S) level. These changes are shown to affect mitochondrial activity and function [41, 42, 43, 44]. However, it is unknown how a change in the O_2_ level will affect mitoNEET reaction with gasotransmitters NO or H_2_S.

Here, we applied a multidisciplinary approach to study how gasotransmitter NO accesses the mitoNEET [2Fe‐2S] cluster and elucidate a possible role of O_2_ in this process. We studied the individual reaction of NO and H_2_S with the mitoNEET cluster in the presence or absence of O_2_. We discovered an explicit NO access site of the [2Fe‐2S] cluster and found that O_2_ or pioglitazone blocked NO access to the mitoNEET [2Fe‐2S] cluster. We found that the reaction of NO with the cluster desensitized the cluster towards reduction by H_2_S. Based on these findings and published data linking mitoNEET expression to mitochondrial activity, we postulate that the mitoNEET [2Fe‐2S] cluster molecular function is to act as an O_2_ and gasotransmitter sensor in hypoxia‐associated mitochondrial signal transduction.

## Materials and methods

### Chemicals

All chemicals were reagent grade and purchased from ThemoFisher Scientific (Waltham, MA, USA), Fisher Scientific, (Loughborough, UK) or Merck, (Rahway, NJ, USA). All anaerobic buffers were prepared a day prior to the experiments and stored in the glovebox overnight to ensure complete removal of dioxygen. H_2_S donor and NO donor reagents were JK‐2 (lithium 2‐(phenylphosphonothioylamino)‐3‐phenylpropanoate) and D‐184 (diethylamine NONOate sodium salt hydrate or DEA NONOate) respectively.

### Cloning, expression and purification of mitoNEET

The *Escherichia coli* codon optimised gene‐encoding soluble form of human mitoNEET (lacking 34 residues at the hydrophobic N‐terminus), and its variants V70W and V70C were purchased from GeneArt (ThermoFisher). The synthetic gene for the soluble wild‐type mitoNEET was in pMQ plasmid and ampicillin resistance. This synthetic gene had a 5′ and 3′ restriction site for KpnI and NcoI, respectively. The codon‐optimized DNA sequence of soluble wild‐type mitoNEET (without restriction sites) is described below. Additionally, the amino acid sequence of soluble wild‐type mitoNEET and its two variants before cloning into pBAD/His C vector are described.

DNA sequence of wild‐type mitoNEET gene used to clone into pBAD: TATGTTAAAGATCATCGTAACAAAGCCATGATCAACCTGCATATCCAGAAAGATAATCCGAAAATCGTGCACGCCTTTGATATGGAAGATCTGGGTGATAAAGCAGTTTATTGTCGTTGTTGGCGCAGCAAAAAGTTTCCGTTTTGTGATGGTGCACATACCAAACATAATGAAGAAACCGGTGATAATGTTGGTCCGCTGATCATCAAAAAGAAAGAAACCTAA.

Wild‐type mitoNEET cloned into pBAD: YVKDHRNKAMINLHIQKDNPKIVHAFDMEDLGDKAVYCRCWRSKKFPFCDGAHTKHNEETGDNVGPLIIKKKET.

mitoNEET‐V1 (V70W) gene cloned into pBAD: YVKDHRNKAMINLHIQKDNPKIVHAFDMEDLGDKAWYCRCWRSKKFPFCDGAHTKHNEETGDNVGPLIIKKKET.

mitoNEET‐V2 (V70C) gene clone into pBAD: YVKDHRNKAMINLHIQKDNPKIVHAFDMEDLGDKACYCRCWRSKKFPFCDGAHTKHNEETGDNVGPLIIKKKET.

Subsequently, the gene encoding each protein was cloned into pBAD/His C (Invitrogen) vector using KpnI and NcoI restriction enzymes (New England Biolabs, Hitchin, UK). After cloning into the pBAD/His C vector, the sequence of the 6‐histidine tag present in the pBAD vector will be added to the N‐terminus of the protein. The wild‐type soluble mitoNEET was cloned in the lab and the presence of the correct insert was confirmed using restriction enzyme double digestion. The variants were cloned into pBAD/His C vector by GeneArt (ThermoFisher). The *E. coli* TOP10 strain (Invitrogen) was transformed with the final expression construct. A glycerol stock of the final *E. coli* cell was prepared and stored at −80 °C freezer. The glycerol stock was used to prepare LB Agar (100 μm ampicillin) plates. A single colony was picked and added to 50‐mL LB media, and the pre‐culture was grown overnight at 37 °C in a shaker (225 r.p.m.). The next day, cells were inoculated into a 2‐L flask containing 500‐mL terrific broth (TB) medium. The culture was grown in a shaker (37 °C, 225 r.p.m.) to an OD@600 nm of 0.6. Subsequently, mitoNEET production was induced by the addition of 0.04%w/v arabinose (final concentration). After 8 h, cells were collected and were pelleted by centrifugation at 4696 x *g* at 20 °C for 20 min. The cell pellet was stored at −80 °C. The pellet was thawed for 30 min at room temperature. Then, cells were resuspended in lysis buffer (50 mm Tris pH 8.0, 300 mm NaCl, 2% Triton, 0.5 mm Dithiothreitol (DTT), 0.5 mm DNAse and 0.5 mm PMSF (phenylmethylsulphonyl fluoride)) with a ratio of 3 mL lysis buffer to 5 mL cells. The cell paste was vortexed, and lysozyme (1 mg·mL^−1^) was prepared in lysis buffer and added to the resuspended cells (1 : 10 V/V). Fully resuspended cells were lysed on ice using a Branson SFX 150 Sonifier (Emerson, Leicester, UK). The conditions were 30%, 10 cycles each 30 s on followed by 10 s off. Each time, 5 mL of resuspended cells were lysed to ensure homogenous lysis. The crude lysate was centrifuged at 4696 x *g* at 4 °C for 40 min to remove cell debris. The supernatant containing soluble proteins was retained and transferred to an anaerobic glovebox (Belle, Weymouth, UK) (O_2_ < 5 ppm). Protein purification under anaerobic conditions was achieved using a gravity column (Thermo Scientific) packed with Ni^2+^ resin (Thermo Scientific). The following buffers were used: Equilibration buffer (50 mm Tris, 300 mm NaCl, 2% triton, 5 mm imidazole, pH 7.5); wash buffer (50 mm Tris, 300 mm NaCl, 2% triton, 0.5 mm DTT, 10 mm imidazole, pH 7.5) and elution buffer (50 mm Tris, 300 mm NaCl, 2% triton, 0.5 mm DTT, 800 mm imidazole, pH 7.5). After purification, PD10 desalting column (Cytiva, Marlborough, MA, USA) was used to exchange the buffer to 50 mm phosphate buffer (pH 7.0) containing 100 mm NaCl and remove imidazole, triton and DTT. The final purified protein was divided into aliquots of 1 mL, flash frozen in liquid nitrogen and stored at −80 °C. Protein purity was assessed by SDS/PAGE, and protein concentration was determined using the bicinchoninic acid (BCA) assay with bovine serum albumin as a standard. The concentration of mitoNEET was between 200 and 270 μm unless otherwise stated. Protein concentrations are the average of three independent measurements ±15% error.

### Expression and purification of hSAND

The *E. coli* codon‐optimized gene for expression of a soluble form of hSAND (lacking N‐terminal hydrophobic domain) was cloned in pBAD/His‐C plasmid, as explained previously [45]. *Escherichia coli* TOP10 cells were transformed with the plasmid encoding hSAND. Expression of hSAND was achieved, as explained for mitoNEET, by the addition of 0.04% W/v arabinose (final concentration). Eight hours after induction, cells were harvested using centrifugation at 4696 x *g* at 20 °C for 20 min. The cell pellet was stored at −80 °C. Cell lysis was performed as explained for mitoNEET. The cell lysate was transferred to an anaerobic glovebox (Belle Technologies) and added to centrifuge tubes. Cell debris was removed by centrifugation at 7810 x *g* and 4 °C for 20 min under aerobic conditions. After centrifugation, the supernatant was retained and immediately transferred to the anaerobic glovebox (O_2_ < 5 ppm). Anaerobic purification was achieved using a gravity column packed with Ni^2+^ resin (Thermo Scientific). The following buffers were used: Equilibration buffer (50 mm Tris, 300 mm NaCl, 2% triton and 5 mm imidazole, pH 7.5); wash buffer (50 mm Tris, 300 mm NaCl, 2% v/v triton, 0.5 mm DTT and 10 mm imidazole, pH 7.5); and elution buffer (50 mm Tris, 300 mm NaCl, 2% v/v triton, 0.5 mm DTT and 500 mm imidazole, pH 7.5). Subsequently, the PD10 desalting column was used to exchange the buffer and remove imidazole, triton and DTT. The final buffer was 50 mm MOPS (3‐(Morpholin‐4‐yl)propane‐1‐sulfonic acid) buffer pH 7.0 containing 100 mM NaCl unless otherwise stated. The sample was flash frozen in liquid nitrogen and stored at −80 °C. The protein purity was confirmed by 10% SDS/PAGE gel, and the protein concentration was measured using the BCA assay. The concentration of hSAND was between 90 and 100 μm unless otherwise stated. Protein concentrations are the average of three independent measurements ±15% error.

### Preparation of different reagents for oxidation/reduction reactions monitored by UV–visible spectrophotometry

To prepare the stock solution of sodium dithionite, NO donor, H_2_S donor or pioglitazone, the desired amount (mg) of their corresponding salt was added to a 1.5‐mL Eppendorf tube and transported into the glovebox. An aliquot of anaerobic buffer (50 mm phosphate buffer, pH 7.0, containing 100 mm NaCl) was added to each vial to reach the desired final concentration, determined based on a specific final reagent to protein and a known protein concentration. To prepare the H_2_O_2_ solution, 500 μL of H_2_O_2_ 30%w/v (Fisher) was transferred into the glovebox *via* a 1.5‐μL Eppendorf vial. The H_2_O_2_ solution was then diluted in anaerobic buffer (50 mm phosphate buffer, pH 7.0, containing 100 mm NaCl).

### General experimental conditions

The proteins and reagent concentrations of samples are provided in the figure legend for each experiment. For all experiments (unless otherwise stated), the buffer was 50 mm phosphate buffer, pH 7.0 and contained 100 mm NaCl. Samples were prepared anaerobically (O_2_ < 5 ppm) in a glovebox (Bele Technology, Weymouth, UK) at room temperature (22 °C) unless otherwise stated. After adding NO donor, H_2_S donor or H_2_O_2_ to the mitoNEET or hSAND solution, samples were incubated for 4 h at room temperature (22 °C) under anaerobic conditions (O_2_ < 5 ppm) in a glovebox (Bele Technology) before subsequent treatment or measurements. To expose mitoNEET or hSAND to O_2_, the anaerobic solutions of the proteins were taken out of the glovebox and incubated under aerobic conditions at room temperature for 4 h. Subsequently, samples were used for further analysis unless otherwise stated. All samples were centrifuged before measurement to remove any possible precipitate interfering with measurement.

### Exposure of proteins to NO or H_2_S

To expose the reduced or oxidized hSAND or mitoNEET to NO or H_2_S, the following components were added in order: 50 μL purified protein solution, phosphate buffer pH 7.0 (an aliquot was added to make up a final volume of 700 μL), 50 μL sodium dithionite (for reduced samples only) and 50 μL NO‐ or H_2_S‐donor solution. When sodium dithionite was added, samples were incubated for 15 min, and then NO‐ or H_2_S‐releasing molecules were added. The samples were incubated for 4 h in the glovebox under anaerobic conditions before analysis using UV–visible spectrophotometry. Five samples were prepared for the NO titration experiments. Initially, 50 μL purified protein solution and phosphate buffer pH 7.0 (an aliquot was added to make up a final volume of 700 μL) were mixed. Subsequently, NO donor (1.12 mm stock prepared in phosphate buffer) was added anaerobically to each sample in varying ratios relative to mitoNEET: 5, 10, 50, 100 or 500 μL, corresponding to a NO donor/mitoNEET ratio of 0, 0.5, 1, 5, 10 or 50 respectively. These samples were incubated anaerobically for 4 h before analysis using UV–visible spectrophotometry. Afterwards, all samples were immediately transferred into a glovebox and incubated overnight to eliminate residual NO. The following day, 50 μL of H_2_S‐donor solution was added to each sample, followed by another 4‐h incubation under anaerobic conditions before UV–visible spectrophotometry analysis. All measurements were repeated two times to confirm reproducibility.

### Exposure of proteins to H_2_O_2_

To expose the reduced or oxidized hSAND or mitoNEET to H_2_O_2_, the following components were added in order: 50 μL purified protein solution, phosphate buffer pH 7.0 (an aliquot was added to make up a final volume of 700 μL), 50 μL sodium dithionite (for reduced samples only) and 50 μL diluted H_2_O_2_ solution. For the reduced sample after adding sodium dithionite, samples were incubated at room temperature under anaerobic conditions for 30 min. In the case of mitoNEET, sodium dithionite was removed under anaerobic conditions (O_2_ < 5 ppm) using a PD10 desalting column, and the formation of the reduced cluster was confirmed using UV–visible spectrophotometry. The samples were then mixed well and incubated for 4 h in the glovebox before analysis using UV–visible spectrophotometry.

### Exposure of proteins to O_2_

To expose oxidized mitoNEET or hSAND to molecular oxygen, the as‐isolated protein was incubated for 4 h in atmospheric conditions. To expose reduced mitoNEET or hSAND to the molecular oxygen, an aliquot of isolated mitoNEET or hSAND was first transported into the anaerobic glovebox. A reaction was made by adding 50 μL purified protein solution, phosphate buffer pH 7.0 (an aliquot was added to make up a final volume of 700 μL) and 50 μL sodium dithionite. Subsequently, the reduced protein was incubated for 4 h under aerobic conditions at room temperature (22 °C), and samples were analysed using UV–visible spectrophotometry.

### Sequential reduction–oxidation mitoNEET

To test the oxidation with NO, H_2_O_2_ or O_2_, and subsequent reduction by H_2_S or sodium dithionite samples were prepared as follows. First, 50 μL of purified mitoNEET was diluted in an aliquot of phosphate buffer pH 7.0 (an aliquot was added to make up a final volume of 700 μL), followed by the addition of 50 μL NO‐donor solution or H_2_O_2_ (NO donor or H_2_O_2_‐to‐mitoNEET ratio of 1). The samples were incubated overnight under anaerobic conditions at room temperature (22 °C) to ensure complete removal of any residual NO in the solution. For oxidation with O_2_, as‐isolated mitoNEET was taken out of the glovebox, and samples were retained under aerobic conditions at room temperature for 4 h. Subsequently, samples were transported back into the anaerobic glovebox and incubated overnight under anaerobic conditions to ensure the removal of any residual O_2_. The next day, 50‐μL H_2_S‐donor solution (to‐mitoNEET ratio of 1) or sodium dithionite (to‐mitoNEET ratio of 1) was added to each sample, and reactions were incubated under anaerobic conditions for 4 h. To reduce samples and then test oxidation, H_2_S donor or (S_2_O_4_)^2−^ was first added, and then O_2_ or NO donor, and all the other conditions were kept the same. Samples were subject to analysis using UV–visible spectrophotometry before and after the addition of reducing and oxidizing agents. In all cases, the total final volume of the sample was 700 μL.

### NO and O_2_ competition studies

To test the competition between NO and O_2_, we tested if H_2_S could reduce the NO‐exposed cluster in the presence of O_2_ and compared that with the control in the absence of O_2_. The control and sample were prepared in parallel. At first, for the control and sample, 50‐μL purified protein solution was added to phosphate buffer pH 7.0 (an aliquot was added to make up a final volume of 700 μL) inside an aerobic glovebox (O_2_ < 5 ppm). Subsequently, the reaction sample in a 1‐mL cuvette was taken out of the anaerobic glovebox (Belle Technology) and left under aerobic conditions for 4 h with gentle shaking to allow slow diffusion of O_2_. The control was incubated under anaerobic conditions (O_2_ < 5 ppm) at the same time. After 4 h, 50 μL of DEA NONOate solution (NO donor) was added to the sample (under aerobic conditions) and control (under anaerobic conditions) to reach a mitoNEET/NO‐donor ratio of 1. The sample and the control were incubated for 4 h under aerobic and anaerobic conditions, respectively. The sample was then transported back into the glovebox. The sample and control were incubated overnight to ensure the removal of any residual O_2_ and NO. The next day, 50‐μL H_2_S‐donor solution was added anaerobically in the glovebox (O_2_ < 5 ppm) to the sample and control to reach a final mitoNEET/H_2_S‐donor ratio of 1. The sample and control were then incubated under anaerobic conditions for 4 h. At each stage, the sample and control were subject to analysis using UV–visible spectrophotometry. In all cases, the total final volume of the sample was 700 μL. Experiments were repeated at least three times on different days to confirm reproducibility.

### Prediction of NO released by DEA NONOate

The measured decomposition rate constant (*k*_dec_) of DEA NONOate at 25 °C and pH 7.0 is approximately 0.0007 s^−1^ [46]. The conditions used in our NO and O_2_ competition studies. The DEA NONOate decomposition is characterized by first‐order release kinetics [47], and thus, for the NO donor (N), we can write:

(1) $$\left[N\right]={\left[N\right]}_{0}e^{\left(-k_{\mathrm{dec}}t\right)}$$

Using this formula, the concentration of DEA NONOate as a function of time is obtained for an initial NO concentration of 16 μm (used in NO and O_2_ competition studies). Each mole of DEA NONOate releases circa 1.5 moles of NO [47]. Hence, the amount of NO released for an initial DEA NONOate concentration of 16 μm is predicted. This amount is much less than 1 μm·s^−1^ at any time point. The NO release rate is defined by the mass conservation equation [47]:

(2) $$\frac{d\left[\mathrm{NO}\right]}{dt}=\mathrm{NO}\;\text{release rate}-4k^{*}{\left[\mathrm{NO}\right]}^{2}\left[O_{2}\right]-\left(k_{L}a/V\right)\left[\mathrm{NO}\right]$$

The second term is the molar rate at which NO reacts with O_2_, and the third term is the molar rate at which NO leaves the solution. Since no stirring was used during the NO reaction in our experiment, the last term is negligible. On the other hand, for the second term describing the NO reaction with O_2_ in air with normal composition, the O_2_ partial pressure is 0.21 atm. Thus, the O_2_ solubility is 254 μm in normal pure water [48]. For a well‐stirred experimental setup at 37 °C, the k* is 2.4 × 10^6^ (m ^−2^S^−1^). Using this k* value (which is an overestimated), the amount of NO reacted with O_2_ at any time is negligible. Therefore, in our setup with no stirring and at 25 °C, the reaction between NO and O_2_ is insignificant (see Results). Consequently, the amount of NO in the solution is approximately equal to the amount of NO generated by the decomposition of DEA NONOate.

### NO trap experiments

A stock solution (21 μm) of *N*,*N*,*N*′,*N*′‐tetramethyl‐p‐phenylenediamine (Sigma Aldrich, St. Louis, MO, USA), the NO‐trap reagent, was prepared under anaerobic conditions by dissolving the compound in the anaerobic phosphate buffer (pH 7.0). The vial was immediately covered with aluminium foil to protect it from light exposure. Separately, a stock solution of DEA NONOate was prepared in phosphate buffer (pH 7.0) under aerobic conditions at the same concentration as the NO trap solution. Immediately, 50 μL of the DEA NONOate stock solution was diluted into 600 μL of phosphate buffer in a plastic cuvette. A total of five samples were prepared, and one control cuvette contained only phosphate buffer. All samples and the control were incubated under aerobic conditions. At time 0 (*t*₀), 50 μL of the NO trap solution was added to the control cuvette and one sample cuvette. Each cuvette was gently shaken to ensure thorough mixing, and the UV–visible absorbance was measured immediately. Subsequent measurements were conducted hourly after incubating samples aerobically: every 60 min, 50 μL of the NO trap solution was added to one additional sample cuvette, mixed by gentle shaking and the UV–visible absorbance spectrum was recorded.

### Exposure to pioglitazone and gasotransmitters

Pioglitazone was added before or after exposure to NO under anaerobic conditions at room temperature (22 °C). First, 50 μL purified protein solution was added to phosphate buffer pH 7.0 (calculated for a total reaction volume of 700 μL), followed by the addition of 50‐μL pioglitazone solution. The sample was incubated for 2 h, and then 50 μL NO‐donor solution was added to the protein and mixed thoroughly. The sample was then incubated overnight under anaerobic conditions (22 °C) to ensure complete removal of unreacted NO. The next day, 50 μL of H_2_S donor was added, and the mixture was incubated for 4 h. In another sample, pioglitazone was added after the addition of DEA NONOate. To this end, after adding DEA NONOate, the sample was incubated in the glovebox under anaerobic conditions (22 °C) overnight for NO exposure and removing any residual NO. The next day, 50‐μL pioglitazone solution was added under anaerobic conditions (22 °C), and the mixture was for 2 h at room temperature under anaerobic conditions. Finally, 50 μL H_2_S‐donor solution was added to the mixture, and the sample was incubated for 4 h in the anaerobic glovebox. UV–visible absorbance spectrum was recorded at each step before and after adding pioglitazone, NO and H_2_S. The NO donor‐, H_2_S donor‐ and pioglitazone‐to‐mitoNEET ratio was 1.

### UV–visible spectrophotometry

To record the UV–visible absorbance spectra, samples were added anaerobically to a 1‐mL disposable plastic cuvette (BRAND™, Willimantic, CT, USA) with a path length of 1 cm and equipped with a silicone lid (BRAND™) to tightly close and prevent oxygen exposure. Each sample was transported outside, and the UV–visible absorbance spectrum was recorded immediately. Measurements were done at room temperature using a PerkinElmer UV/Vis Lambda 365 Instrument (Shelton, CT, USA). Spectra were recorded from 300 to 700 nm, which is the wavelength window used by others to study mitoNEET [32, 38]. Measurements were done at room temperature (22 °C) under aerobic conditions.

### Preparation of samples for liquid chromatography–mass spectrometry analysis (LC–MS) of 3՛‐deoxy‐3՛,4՛‐didehydro‐CTP (ddhCTP) formation

Anaerobic buffer, 50 mm MOPS, pH 7.0, containing 100 mm NaCl, was used to prepare stock solutions of SAM, cytidine triphosphate (CTP), H_2_S donor and sodium dithionite under anaerobic conditions in a glovebox (Belle Technology (O_2_ < 5 ppm)). An aliquot of chemicals was transported into the glovebox and mixed with the buffer under anaerobic conditions. The buffer was prepared a day prior to the experiments and stored in the glovebox overnight to ensure complete anaerobicity. Four samples were prepared. The complete reaction had 500 μL of purified hSAND solution, 20 μL SAM chloride (S‐(5′‐adenosyl)‐l‐methionine‐(S‐methyl‐13C) chloride), 20 μL CTP (cytidine 5′‐triphosphate disodium salt) and 20 μL Na_2_S_2_O_4_ (sodium dithionite). The anaerobic MOPS buffer pH 7.0 was added to reach a final volume of 660 μL. A negative control sample was prepared. In the negative control, hSAND was not added, and it was replaced with buffer and NADH. The reaction mixture was incubated anaerobically overnight. The following day, the sample was centrifuged at 16250 x *g* for 3 min. Then, 450 μL of the reaction was added to a 0.5‐mL Amicon Ultra Centrifugal Filters 3 kDa (Merck) and centrifuged at 16250 x *g* for 30 min. The resulting flowthrough was then carefully transferred to an HPLC low volume (300 μl) recovery vial (Fisher Scientific) and sealed tightly with a polypropylene cap (Fisher Scientific). Each experiment was repeated at least two times with different batches of protein to test reproducibility.

To test the activity of hSAND exposed to H_2_S, we prepared three samples. Sample 1, hSAND exposed to H_2_S only: 200 μL of 140 μm purified hSAND solution was mixed with 10 μL of stock solution of H_2_S donor, then the mixture was incubated under anaerobic condition for 2 h, followed by the added 10 μL SAM, 10 μL CTP and 10 μL of anaerobic MOPS buffer pH 7.0 to reach a final volume of 250 μL. Sample 2, hSAND exposed to H_2_S and (S_2_O_4_)^2−^: 200 μL of 140 μm purified hSAND solution was exposed to 10 μL stock solution of H_2_S donor for 2 h. Next, 10 μL of sodium dithionite was added, followed by the addition of 10 μL of SAM and 10 μL of CTP. Sample 3, the control hSAND sample not exposed to H_2_S: 200 μL of 140 μm purified hSAND solution was mixed with 10 μL of sodium dithionite, 10 μL of SAM and 10 μL of CTP and 10 μL of buffer anaerobic MOPS, buffer pH 7.0 to reach a final volume of 250 μL. Samples were incubated overnight, subject to filtration using 3 kDa Amicon ultracentrifugal filter to remove proteins and the flowthrough was used to measure the formation of ddhCTP from CTP. Experiments were repeated at least two times to confirm reproducibility.

### LC–MS

Measurements were performed using an Agilent InfinityLab G6160A LC/MSD iQ mass spectrometer equipped with [Agilent 1260 Infinity II] liquid chromatography system. Coumn was Agilent ZORBAX RR HILIC Plus Column, 2.1 x 100 mm, 3.5 µm. Instrument control and data processing were performed using openlab cds (Agilent Technologies, Santa Clara, CA, USA). The system was calibrated on the day of the analysis. Electrospray source conditions were adjusted to maximize sensitivity, and the detection mode was set to detect negative (−) ions. For each run, 50 μL of solution was injected with a flow rate of 0.2 mL·min^−1^ and an oven temperature of 45 °C:

- Buffer A: 90 vol.% MeCN (acetonitrile) (Fisher, 99.9%): 10 vol.% LC–MS grade water (Fisher), 20 mm ammonium acetate, pH 7.4–7.5.
- Buffer B: 10 vol.% LC–MS grade water (Fisher), 20 mm ammonium acetate, pH 7.4–7.5.
- Column:
- Flow rate: 0.2 mL·min^−1^
- Gradient: [0–1 min]: 100 : 0 (A : B); [1–10 min] 100 : 0 (A : B) linearly changed to 10 : 90 (A : B); [10–15 min] 10 : 90 (A : B); [15 : 17] linearly changed to 100 : 0 (A : B) and [17–35 min] 100–0 (A : B).

Analysis of the LC–MS data was performed using openlab cds software.

### Preparation of sample for electron paramagnetic resonance (EPR) spectroscopy

Anaerobic buffer (50 mm phosphate, pH 7.0, 100 mm NaCl) was used to prepare different reagents used in experiments. All reagents were transported into the glovebox and mixed with the buffer under anaerobic conditions. The buffer was prepared a day prior to the experiments and stored in the glovebox overnight to ensure complete removal of dioxygen. For NO and H_2_S exposed, the NO or H_2_S donor was dissolved in buffer and immediately added to the solutions. To prepare the samples, components were added in the following order: 150 μL purified protein solution, phosphate buffer (an aliquot added to make up a total volume of 200 μL), 20 μL (S_2_O_4_)^2−^ (for reduced samples only) and 20 μL reagent solution. The molecular ratio of NO donor, H_2_S donor or (S_2_O_4_)^2−^ to protein was 1. Samples were incubated overnight under anaerobic conditions. Next, they were transferred into 3 mm ID, 4 mm OD or 1.1 mm ID, and 1.6 mm OD clear fused quartz EPR tubes (Wilmad 707‐SQ‐250M and Wilmad WG‐221T‐RB, respectively). Samples were transferred to the outside of the glovebox and frozen immediately in liquid nitrogen.

### Additional methods

Details of EPR measurements and molecular dynamics (MD) simulations are available in Data S1.

## Results

### NO degrades hSAND cluster

**Fig. 2:**
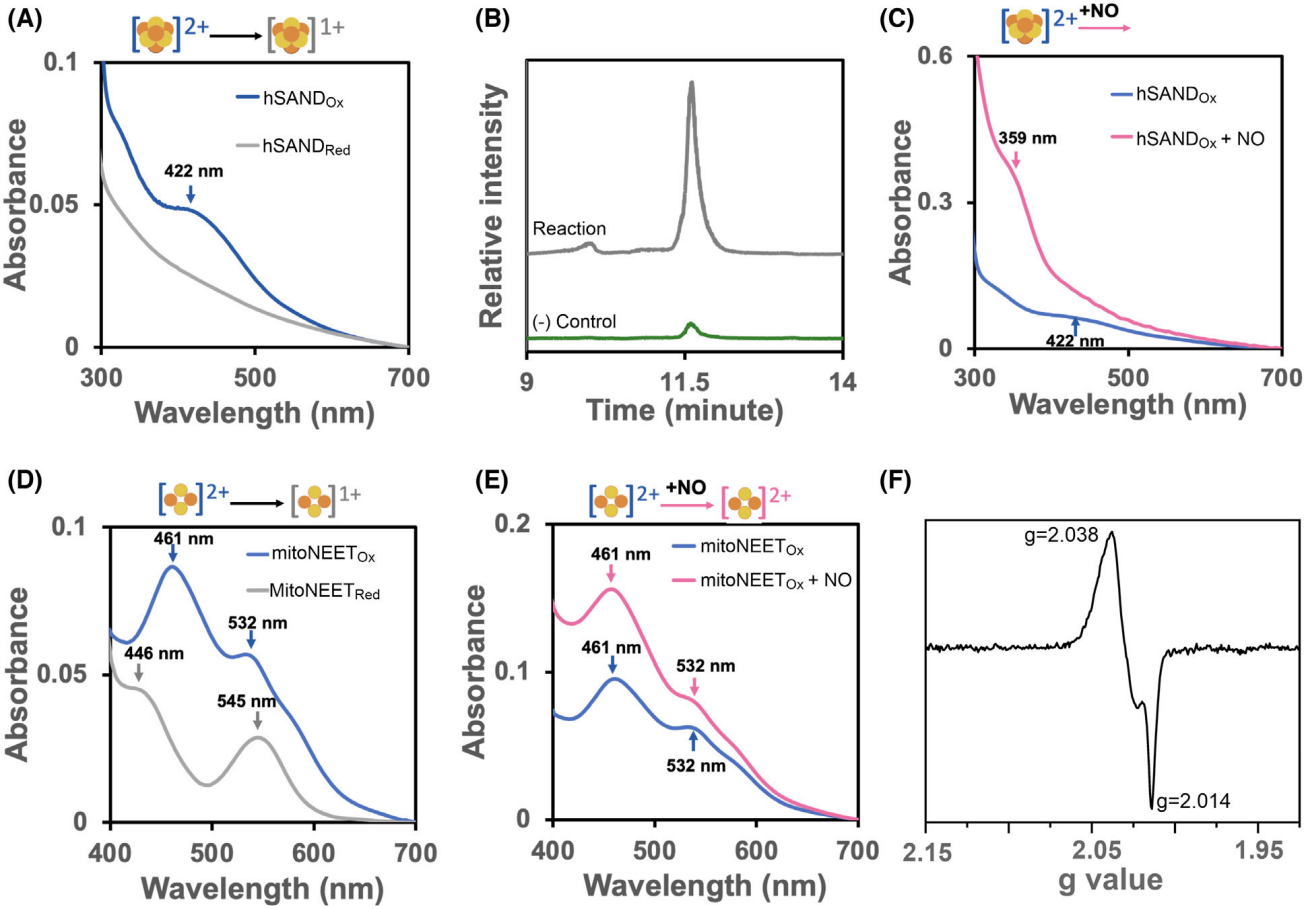
The SAND [4Fe‐4S] cluster and mitoNEET [2Fe‐2S] cluster react with NO differently. (A) UV–visible absorbance spectrum of hSAND (5 ± 1 μm) [4Fe‐4S] cluster before (hSAND_Ox_) (blue) or after (hSAND_Red_) (grey) reduction (30 min) by sodium dithionite ((S_2_O_4_)^2−^) (5 μm). (B) LC–MS analysis of the formation of ddhCTP. The graph shows the extracted ion chromatograph (EIC) of ddhCTP with a [M‐H]^−1^ m/z of 464.0. The reaction contained hSAND (53 μm), (S_2_O_4_)^2−^ (5.3 mm), SAM (21.2 mm) and CTP (5.3 mm). The negative control did not have hSAND. (C) The reaction of oxidized hSAND [4Fe‐4S] cluster (8 μm) with NO released (4 h) from DEA NONOate (8 mm). (D) The UV–visible absorbance spectrum of mitoNEET [2Fe‐2S] cluster (10 ± 2.65 μm) before (mitoNEET_Ox_) (blue) and after (mitoNEET_Red_) (grey) reduction (30 min) by sodium dithionite ((S_2_O_4_)^2−^) (10 μm). (E) The reaction of oxidized mitoNEET [2Fe‐2S]^2+^ cluster (10 μm) with NO released (4 h) from NONOate (1 mm). (F) CW‐EPR spectrum at T = 40 K of mitoNEET [2Fe‐2S]^2+^ cluster (200 μm) oxidized with NO released from DEA NONOate (215 μm), incubated overnight under anaerobic conditions. (A–E) Were repeated three times, and (F) was repeated two times to confirm reproducibility. Buffers were (A, C–F) phosphate 50 mm, 100 mm NaCl, pH 7.0 and (B) MOPS 50 mm, 100 mm NaCl, pH 7.0. All samples were prepared at room temperature (~ 22 °C) under anaerobic conditions (O_2_ < 5 ppm).

The reaction of O_2_, H_2_O_2_ and NO with most [Fe‐S] proteins leads to the degradation of the cluster. To have a system for comparing the degradation reaction with the sensory reaction, we first investigated the reaction of O_2_, H_2_O_2_ and NO with the hSAND [4Fe‐4S] cluster, which has not been studied. We first overexpressed and purified the hSAND soluble form lacking the N‐terminus hydrophobic membrane anchoring domain. Next, we used a combination of spectroscopic methods and LC–MS and investigated the effect of gasotransmitter NO on the hSAND [4Fe‐4S] cluster. We compared the results with those we obtained from the effect of other oxidizing agents, namely, dissolved O_2_ gas and H_2_O_2_. The purified hSAND was in the oxidized state (Fig. 2A), and the addition of sodium dithionite reduced the cluster as observed by the disappearance of the UV–visible absorbance peak of the [4Fe‐4S]^2+^ cluster between 400 and 450 nm (Fig. 2A). The enzyme with the reduced cluster was active and capable of converting CTP to its nucleotide analogue ddhCTP [49, 50], as measured by LC–MS (Fig. 2B). Exposure of oxidized [4Fe‐4S]^2+^ cluster to O_2_ (Fig. S1A) or H_2_O_2_ (Fig. S1B) led to the disappearance of the absorbance peak of the [4Fe‐4S]^2+^ cluster, suggesting degradation of the cluster consistent with our previous observation of O_2_‐mediated degradation of the fungal SAND cluster [51]. Similarly, the NO released by the NO‐donor agent dimethylamine NONOate (DEA NONOate) under anaerobic conditions degrades the hSAND cluster. The exposure of the oxidized (Fig. 2C) or reduced (Fig. S1C) hSAND to NO led to the disappearance of the [4Fe‐4S]^2+^ cluster absorbance peak at 400–450 nm and the appearance of a new peak with a maximum absorbance of approximately 359 nm. The observation of this peak is somewhat similar to that reported previously for iron‐nitrosyl complexes [52]. While NO‐exposed enzyme completely lost its ability to catalyse the transformation of CTP to ddhCTP, the O_2_‐ and H_2_O_2_‐exposed enzymes retained approximately 30% and 10% of their activities, respectively (Fig. S1D). These data confirmed that NO, O_2_ and H_2_O_2_ degrade the hSAND cluster and that NO degradation is more severe. Therefore, NO reacts with the [4Fe‐4S] cluster and degrades it, generating iron‐nitrosyl species, possibly like those observed by NO‐mediated degradation of [4Fe‐4S] cluster of NO sensory proteins [53].

### mitoNEET [2Fe‐2S] cluster senses NO in a specific way

Next, we purified a soluble form of mitoNEET lacking the N‐terminus hydrophobic domain and studied how NO, O_2_ or H_2_O_2_ react with its cluster. The individual reaction of O_2_, H_*2*_O_2_ or NO with mitoNEET [2Fe‐2S] cluster has been studied extensively previously [31, 32, 33, 34, 35, 36, 37, 38, 39, 40]. We repeated these experiments to understand the system and be able to further investigate the molecular mechanism of the reaction of NO and the interplay among NO, O_2_ and H_2_S reactions with the mitoNEET [2Fe‐2S] cluster, unlike previous works [31, 32, 33, 34, 35, 36, 37, 38, 39, 40]. The purified mitoNEET showed two absorbance peaks at 461 and 532 nm, typical of the oxidized [2Fe‐2S]^2+^ clusters and the cluster was reduced by sodium dithionite showing two absorbance peaks at 446 and 545 nm (Fig. 2D). For subsequent studies with mitoNEET, we chose the more physiologically relevant phosphate buffer (pH 7.0) since, unlike MOPS buffer, it did not affect the mitoNEET‐oxidized cluster (Fig. S2A). Unlike hSAND, whose cluster was degraded by NO, O_2_ or H_2_O_2_, the mitoNEET [2Fe‐2S] cluster was not and reacted differently with each molecule. The exposure of oxidized mitoNEET [2Fe‐2S]^2+^ cluster to H_2_O_2_ or O_2_ did not affect the cluster (Fig. S2B,C). Both O_2_ and H_2_O_2_ were able to oxidize the reduced [2Fe‐2S]^1+^ cluster (Fig. S2D,E). Exposure of the oxidized (Fig. 2E) or reduced (Fig. S2F) cluster to NO released from NO donor generated an oxidized form of [2Fe‐2S]^2+^ cluster with a higher absorbance than that of the as‐isolated oxidized protein. The addition of sodium dithionite reduced the NO‐exposed cluster, generating an absorbance spectrum identical to that of the reduced cluster not exposed to NO (Fig. S2G). Therefore, NO oxidizes the [2Fe‐2S] cluster but does not degrade it unlike what we observed for hSAND. The ability of the mitoNEET cluster (midpoint redox potential of +35 mV) to be oxidized by NO is consistent with the most cited standard potential value of +390 mV (1 m vs. NHE) for the NO/^3^NO^−^ couple [54]. The increase in the absorbance of the NO‐exposed cluster (Fig. 2E), as compared to the O_2_‐oxidized cluster (Fig. S2D), may suggest possible coordination of NO to the cluster as reported by others for the NEET family of proteins [38, 39, 40, 55]. To test this possibility, we exposed the oxidized [2Fe‐2S]^2+^ cluster to NO and performed continuous‐wave (CW) X‐band EPR spectroscopy measurements (Fig. S2H). To prevent any background signal from NO, samples were prepared by incubating mitoNEET and NO donor (the DEA NONOate) in a one‐to‐one ratio and overnight incubation in the glovebox (Data S1) to allow removal of any excess NO. EPR measurements (Fig. 2F) revealed the presence of a signal identical to that reported for iron‐nitrosyls coordinated with cysteine residues of a protein [56, 57]. The percentage of iron‐nitrosyl species, as determined by comparison of the relative CW‐EPR signal intensities, was a fraction (~ 5%) of the total reduced cluster. When we exposed the oxidize [2Fe‐2S]^2+^ cluster to different DEA NONOate‐to‐protein ratios, at the ratio above 50, we observed a significant increase in the UV–visible absorbance spectrum of the cluster (Fig. S2I). Therefore, mitoNEET [2Fe‐2S] cluster reacts with NO in a specific way.

### Discovery of an NO access site to mitoNEET [2Fe‐2S] cluster

**Fig. 3:**
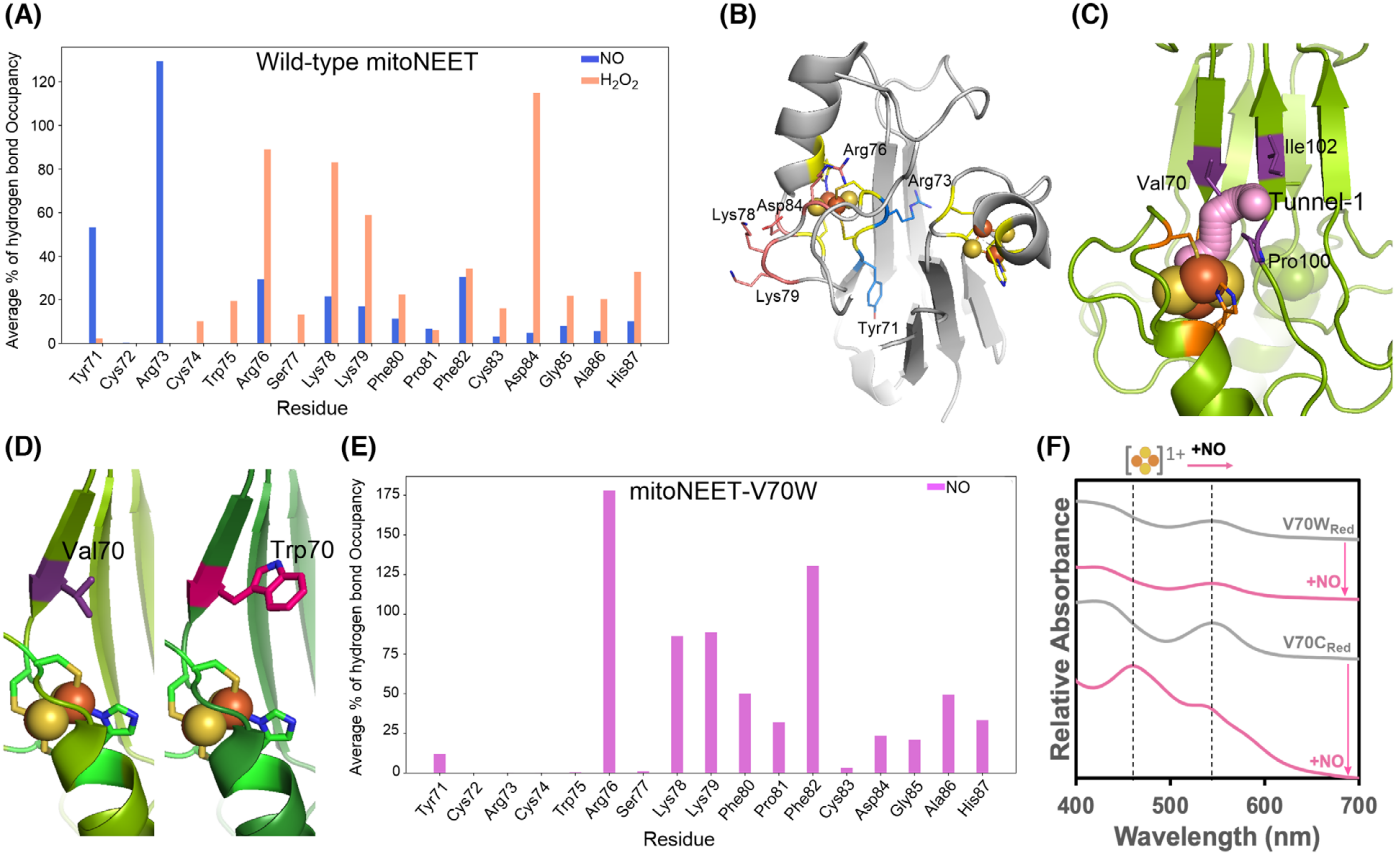
NO reaches the mitoNEET [2Fe‐2S] cluster through an explicit access site. (A) The average % of hydrogen bond occupancy with the residues within 3.5 Å of the [2Fe‐2S] cluster. (B) The structure (PDB code 2QH7) shows residues at the dimer interface interacting with NO (blue) and the main surface residues interacting with H_2_O_2_ (salmon). (C) Schematic presentation of the predicted tunnel 1 with the shortest distance to the cluster. (D) A comparison of the WT and V70W mitoNEET structures suggests that tryptophane covers, at least partially, NO access to the cluster through tunnel 1. (E) The average % of hydrogen bonds formed between NO molecule and individual residues of the mitoNEET‐V70W variant during the last 50 ns of the NO‐diffusion simulation repeats. (A, E) Hydrogen bonds were identified by using the cut‐off distance of 3.5 Å between hydrogen bond donor/acceptor atoms and a bond angle cut‐off of 120°. (F) NO cannot oxidize the reduced [2Fe‐2S]^1+^ cluster of the V70W variant (14 μm), but it oxidizes that of the V70C variant (25 μm). Oxidized mitoNEET was reduced with an equivalent amount of dithionite ((S_2_O_4_)^2−^) and then subject to NO reaction. Proteins were incubated with the NO‐donor agent for 4 h under anaerobic conditions at room temperature (~ 22 °C). The difference between V70W and V70C variants confirms that all dithionite is consumed and does not affect NO oxidation. The ratio of the NO‐donor concentration to that of mitoNEET was 1. Buffer was phosphate 50 mm, 100 mm NaCl, pH 7.

It is unknown how NO reaches the mitoNEET cluster, and previous works have not explored this mechanism [31, 32, 33, 34, 35, 36, 37, 38, 39, 40]. To gain insight into this mechanism, we first used MD simulations to study the interaction of NO and H_2_O_2_ with mitoNEET (Data S1). The average backbone RMSD (root‐mean‐square deviation) values of the protein for 50 ns were less than 2 Å (Fig. S3A), indicating that the system is in a thermal equilibrium. Next, we compared the root‐mean‐square fluctuation (RMSF) of C‐α atoms to assess the protein flexibility in the presence of H_2_O_2_ or NO (Fig. S3B). Protein flexibility in the presence of H_2_O_2_ or NO is similar, with average RMSF values being 0.62 ± 0.37 or 0.6 ± 0.34, respectively. Analysis of the interaction between NO or H_2_O_2_ molecules with the mitoNEET residues located in the vicinity of the [2Fe‐2S] cluster (Fig. 3A and Table S1) showed that H_2_O_2_ largely forms stable hydrogen bonds with the side chains of residues on the protein surface such as Arg76, Lys78, Lys79 and Asp84; while NO forms hydrogen bonds with residues at the interface of the dimer, namely, Tyr71 and Arg73 (Fig. 3B). Thus, while NO diffuses into the protein shell, H_2_O_2_ cannot. Using the caver web 1.0 server, we identified 19 possible (Fig. S3C) NO access tunnels. Most of these tunnels are formed at the interface between two mitoNEET monomers, but tunnel 1 entry site is located directly on the surface at the top of the cluster (Fig. 3C). This short tunnel is notable because it enables direct access to the cluster (Fig. 3C). A total of 19 amino acid residues are involved in the construction of tunnel 1 (Table S2). Four key residues of the entry site of the tunnel, namely, Val70, Pro100, Leu101 and Ile102, cannot participate in hydrogen bonding with NO, thus allowing it accessibility to the [2Fe‐2S] cluster. Consistently, the NO trajectory in the MD simulations shows direct movement towards the cluster through this entry site (Fig. S4A), while for H_2_O_2_, there is no direct path due to the multiple hydrogen bonds it forms with surface residues (Fig. S4B). Additionally, the results of MD calculations predict that NO remains near the cluster for a significantly longer period than H_2_O_2_. The survival probability, defined as the fraction of ligands that remain intact over time, of H_2_O_2_ drops to 0% within just 5 ns, while NO maintains a survival probability of 86% even after 20 ns (Fig. S4C). These findings suggest a specific NO entry site to the mitoNEET [2Fe‐2S] cluster. To validate the result of the predictions, we created two variants. We replaced the highly conserved Val70 of the entry site with the bulky residue tryptophane (mitoNEET‐V70W), hypothesizing that this mutation will cap the NO entry site, abolishing NO access to the cluster. In the second variant, Val70 was replaced with a cysteine (mitoNEET‐V70C) to further facilitate NO access and reaction. The predicted structure of V70W variant shows that tryptophane covers the entry site, possibly blocking NO access (Fig. 3D), while in the V70C variant, the entry site was accessible (Fig. S4D). Analysis of the interaction of NO with mitoNEET‐V70W (Fig. 3E) and ‐V70C (Fig. S4E) residues located in the vicinity of the [2Fe‐2S] cluster revealed that V70W mutation completely abolished the NO interaction with Tyr71 and Arg73 at the interface of two mitoNEET subunits (Fig. 3E), while V70C mutation slightly increased the interactions as compared to wild‐type protein (Fig. S4E). This analysis suggests that in the V70W variant, NO cannot diffuse into the protein. Next, both variants of mitoNEET were purified and characterized. The reduced and oxidized variants have a UV–visible absorbance spectrum the same as the reduced and oxidized wild‐type (WT) proteins, respectively (Fig. S5A,B). Additionally, EPR spectroscopy confirmed that both clusters were reduced the same as the wild‐type mitoNEET [2Fe‐2S] cluster (Fig. S5C,D). Therefore, neither mutation affected the [2Fe‐2S] cluster. Subsequently, we studied the reaction of NO with the reduced or oxidized clusters. NO was unable to oxidize the reduced [2Fe‐2S]^1+^ cluster of the V70W variant (Fig. 3F), unlike what we observed for wild‐type mitoNEET. In contrast, NO was able to reduce the [2Fe‐2S]^1+^ cluster of the V70C variant (Fig. 3F). Additionally, exposure of the oxidized mitoNEET‐V70W variant to NO did not increase the absorbance (Fig. S5E), but that of oxidized mitoNEET‐V70C variant did (Fig. S5F), similar to what we observed for wild‐type protein (Fig. 2E). These findings confirm that the V70W mutation blocked NO access to the cluster. In summary, MD simulations and mutagenesis studies confirm that NO diffuses towards the mitoNEET [2Fe‐2S] cluster through an explicit NO tunnel with an entry site on the surface near the cluster.

### Gasotransmitter H_2_S reduces mitoNEET [2Fe‐2S] cluster

**Fig. 4:**
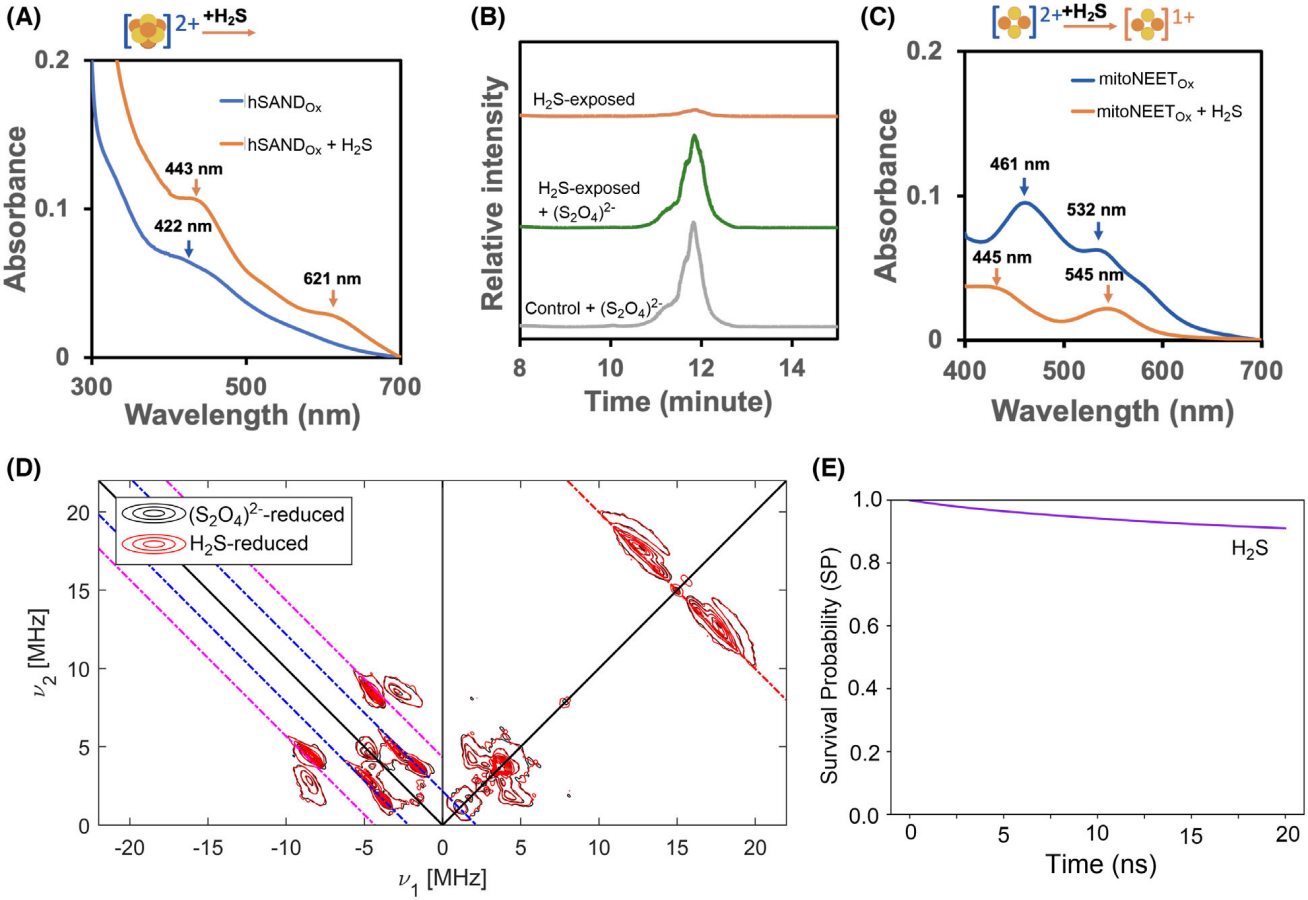
Gasotransmitter H_2_S reduces the mitoNEET [2Fe‐2S] cluster. (A) The reaction of oxidized hSAND (8 μm) with H_2_S (H_2_S donor 3.6 mm) (4 h). (B) The hSAND [4Fe‐4S] cluster is catalytically active after exposure to H_2_S (4 h). The graph shows the extracted ion chromatogram of ddhCTP ([M‐H]^−1^ m/z of 464.0) generated by hSAND exposed to H_2_S only (orange), to H_2_S and then reduced by dithionite ((S_2_O_4_)^2−^) (green) or reduced by (S_2_O_4_)^2−^ only (grey). The SAM, CTP and (S_2_O_4_)^2−^ concentrations were 44.8, 11.2 and 11.2 mm, respectively, and those of hSAND and H_2_S donor were 112 μm. (C) H_2_S reduces the oxidized mitoNEET [2Fe‐2S]^2+^ cluster. mitoNEET (10 μm) was incubated with H_2_S donor (10 m) for 4 h, and the spectra were recorded before and after. (D) HYSCORE spectra at 10 K of dithionite‐reduced (black contour lines) and H_2_S‐reduced (red contour lines) mitoNEET [2Fe‐2S] cluster. The red, blue and magenta dashed lines designate the ^1^H, ^14^N single‐quantum and ^14^N double‐quantum frequencies respectively. The concentrations of mitoNEET, H_2_S donor and (S_2_O_4_)^2−^ were 200, 430 and 215 μm respectively. (E) The survival probability of H_2_S interaction with mitoNEET. (A–C) All experiments were repeated three times, and (D) the experiment was repeated two times to confirm reproducibility. Buffer was (A, C) phosphate 50 mm, 100 mm NaCl, pH 7.0 or (B) MOPS 50 mm, 100 mm NaCl, pH 7.0. (A–D) Samples were prepared at room temperature (~ 22 °C) under anaerobic conditions.

While the reaction of biological thiols such as reduced glutathione (GSH) or cysteine with mitoNEET has been studied [35], the reaction of gasotransmitter H_2_S with mitoNEET cluster and other [Fe‐S] proteins is unknown. Therefore, we tested how H_2_S can affect hSAND and mitoNEET redox state. We first tested the reduction of hSAND [4Fe‐4S] cluster by H_2_S. The redox potential of the [4Fe‐4S] cluster in some radical‐SAM enzymes is predicted to fall in the range of −500 and −450 mV [16], and the two‐electron redox potential of H_2_S is +0.17 mV at pH 7.0 [58]. These redox potentials suggest that H_2_S is unlikely to reduce the [4Fe‐4S] cluster of hSAND. Consistently, we observed that H_2_S did not reduce the hSAND cluster. When the oxidized hSAND was exposed to H_2_S, the absorbance peak of the [4Fe‐4S]^2+^ cluster changed (Fig. 4A). However, this change was not observed when the reduced protein was exposed to H_2_S (Fig. S6A). The CW EPR measurement showed that H_2_S did not reduce the [4Fe‐4S]^2+^ cluster (Fig. S6B). This observation was further confirmed by LC–MS analysis of ddhCTP formation showing that the H_2_S‐exposed enzyme was unable to catalyse transformation of CTP to ddhCTP (Fig. 4B).

On the other hand, the midpoint redox potential of mitoNEET is measured to be +35 mV (at pH 7.5) [59], suggesting that it can be reduced by H_2_S. Consistently, we found that exposure of the oxidized mitoNEET cluster to H_2_S donor generated the reduced [2Fe‐2S]^1+^ cluster with absorbance peaks at 446 and 545 nm (Fig. 4C). When mitoNEET was first reduced by sodium dithionite ((S_2_O_4_)^2−^), it was not further reduced by the addition of H_2_S. The UV–visible absorbance spectra of mitoNEET reduced by H_2_S released from H_2_S donor or sodium dithionite were similar (Fig. S6C). EPR spectroscopy confirmed that the H_2_S‐ and dithionite‐reduced [2Fe‐2S]^1+^ clusters are structurally similar. The CW measurements showed that both samples were reduced (Fig. S6D). Both spectra showed characteristic features of the dipolar interaction between the electron spins of the two adjacent reduced clusters in the dimeric unit of mitoNEET, as described previously [60, 61]. These features were less prominent in the spectrum of the H_2_S‐reduced sample compared to the one reduced by dithionite, consistent with a lower spin count (~ 70%). Hyperfine sublevel correlation (HYSCORE) measurements confirmed that the environment of the [2Fe‐2S] cluster was unchanged. The major peaks in the HYSCORE spectra correspond to those previously assigned to the [2Fe‐2S](Cys)_3_(His) cluster in rat mitoNEET, including those arising from the nitrogen ligand of the histidine and are the same for dithionite and H_2_S reduced cluster (Fig. 4D) [61]. To get further insights into the potential diffusion of H_2_S into the mitoNEET structure, we performed MD simulations. Like NO, the backbone RMSD values of around 2 Å relative to the initial crystal geometry (Fig. S6E) indicate thermal structural stability and minimal conformational changes in the system. The RMSF of C‐α atoms in the presence of H_2_S (Fig. S6F) revealed that the protein flexibility is like that in the presence of NO. Similar to NO and unlike H_2_O_2_, the highest hydrogen bond occupancy was observed with the side chain of Arg73 located in the interface between the dimers (Fig. S6G). Additionally, the survival probability of H_2_S remains around 90% after 20 ns (Fig. 4E) suggesting stronger interaction of H_2_S as compared to NO, whose survival probability was 86%. We observed that H_2_S reduced the oxidized cluster of the V70W and V70C variants of mitoNEET (Fig. S6H,I). This observation suggests that H_2_S either reaches the cluster through other predicted tunnels or does not need to react directly with the cluster to reduce it.

### Pioglitazone blocks NO access

**Fig. 5:**
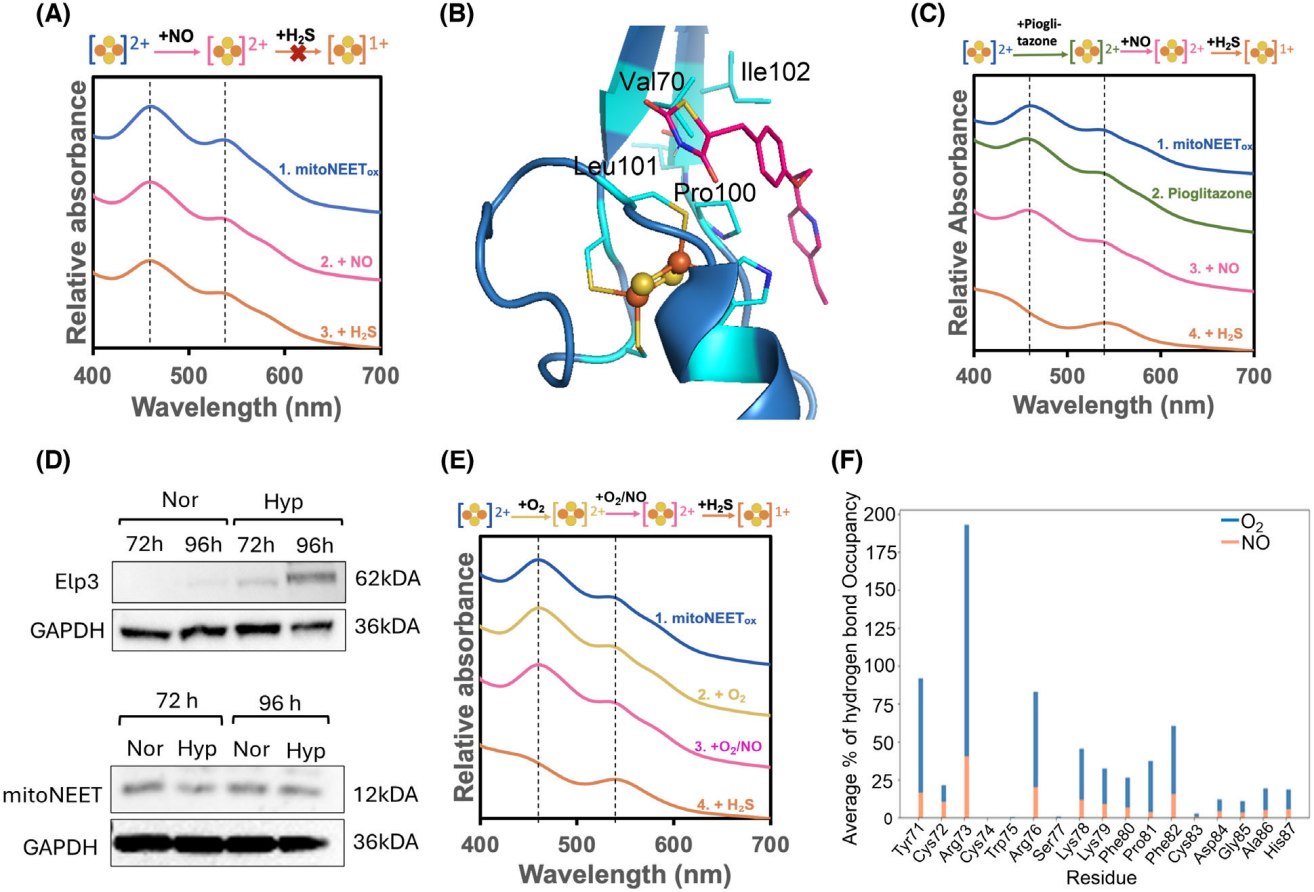
O_2_ and pioglitazone block the NO access to the mitoNEET [2Fe‐2S]^2+^ cluster. (A) Exposure of the anaerobically NO‐exposed (overnight) oxidized mitoNEET [2Fe‐2S]^2+^ cluster to H_2_S released from H_2_S donor (4 h under anaerobic conditions) does not reduce the cluster. (B) Docking of pioglitazone using the structure of mitoNEET (PDB Code: 3REE) predicts its binding to the residues forming the NO entry site, residues Val70, Pro100, Leu101 and Ile102. (C) The addition of pioglitazone before NO exposure (overnight under anaerobic conditions) restores the ability of H_2_S released from H_2_S donor (4 h under anaerobic conditions) to reduce the NO‐exposed mitoNEET [2Fe‐2S]^2+^ cluster. (D) Western blot analysis of the expression of Elp3 (62 kDa) ([4Fe‐4S](Cys)_3_ cluster) and mitoNEET (15 kDa) in T_effs_ under hypoxic (Hyp) or normoxic (Nor) conditions. GAPDH was used as a loading control. (E) H_2_S reduces aerobically NO‐exposed (O_2_/NO‐exposed cluster) mitoNEET. The mitoNEET cluster was exposed to NO (4 h) under aerobic conditions (O_2_/NO‐exposed cluster) and subsequently incubated under anaerobic conditions (overnight) and then exposed to H_2_S (4 h under anaerobic conditions). (F) The average % of hydrogen bond occupancy with the residues near the [2Fe‐2S] cluster. The graph shows O_2_ occupancy in the presence of NO (blue) and NO occupancy in the presence of O_2_ (Salmon). Hydrogen bonds were identified by using the cut‐off distance of 3.5 Å between hydrogen bond donor/acceptor atoms and a bond angle cut‐off of 120°. The concentrations of mitoNEET were (A, C) 16 μm or (E) 19 μm. The ratio of (S_2_O_4_)^2−^, DEA NONOate (NO donor), H_2_S donor or pioglitazone to mitoNEET was 1. All experiments were repeated at least three times to confirm reproducibility. Buffer was phosphate 50 mm, 100 mm NaCl, pH 7. (A, C, E) Samples were prepared at room temperature (~ 22 °C).

It is unknown if the reaction of NO with the mitoNEET [2Fe‐2S] cluster affects its ability to be reduced by biologically relevant reducing agents such as H_2_S. To address this question, we tested if H_2_S can reduce the mitoNEET [2Fe‐2S]^2+^ cluster after its exposure to O_2_ or NO, the latter in the presence of very low O_2_ level (circa 5 ppm) (approximately 7 nm). We found that H_2_S reduced the O_2_‐oxidized cluster (Fig. S7A) like sodium dithionite (Fig. S7B), but it did not reduce the NO‐exposed cluster when the O_2_ level was low (Fig. 5A), unlike sodium dithionite (Fig. S2G). The ability of H_2_S to reduce the cluster was dependent on the amount of NO used. At a NO‐to‐mitoNEET ratio of 0.5, only about half the clusters could be reduced as the absorbance of the oxidized cluster decreased circa 50% (Fig. S7C). At a NO‐to‐mitoNEET ratio of 1 or more, H_2_S could not reduce any of the clusters, and the absorbance of the NO‐exposed oxidized cluster did not change after exposure to H_2_S (Fig. S7C). Conversely, NO was able to oxidize the H_2_S‐reduced cluster (Fig. S7D). These findings confirm that when the O_2_ level is low, the reaction of NO with mitoNEET [2Fe‐2S] cluster desensitizes it towards reduction by gasotransmitter H_2_S. It is known that type 2 diabetic drug pioglitazone interacts with mitoNEET and attenuates mitochondrial oxidative damage [62, 63]. However, the exact mechanism of pioglitazone action is unknown. We hypothesized that TZD ligands like pioglitazone may protect mitoNEET against oxidation by NO and, therefore prevent desensitization towards H_2_S reduction. Previous structural studies revealed that TZD ligands like sulphonamide (Fig. S7E) bind on the surface of the mitoNEET cluster, interacting with amino acid residues we predicted to form the entry site of tunnel 1. Similarly, our docking studies using pioglitazone (Fig. 5B and Fig. S7F) showed that this ligand also binds to the surface of mitoNEET and interacts with Val70, Pro100, Leu101 and Ile102, the residues forming the NO entry site. Therefore, pioglitazone may block the NO access and thereby prevent the reaction of NO with the mitoNEET [2Fe‐2S] cluster. To test this possibility, we added pioglitazone to mitoNEET (1 : 1 ratio) before (Fig. 5C) or after (Fig. S7G) exposure to NO under abnormally low O_2_ levels (5 ppm). mitoNEET was incubated with the NO‐donor reagent. After overnight incubation to ensure the removal of NO gas from the solution, H_2_S donor was added. We observed that under these conditions, H_2_S was able to reduce the cluster (Fig. 5C), unlike in the absence of pioglitazone (Fig. 5A). Therefore, pioglitazone blocked the NO diffusion towards the cluster and thereby preventing the cluster desensitization towards reduction by H_2_S.

### O_2_ blocks NO access

The link between hypoxia and mitochondrial dysfunction is established [44, 64], and mitoNEET oxidation state is linked to voltage gating function of VDAC1 and mitochondrial function [25]. Additionally, hypoxia affects mitochondrial [Fe‐S] cluster biogenesis and causes VDAC1 cleavage [65]. However, it is unknown if normoxic conditions would protect the mitoNEET [2Fe‐2S] cluster against NO oxidation and desensitization towards reduction by H_2_S. To this end, we first studied how the O_2_ level would affect the mitoNEET cellular level. Interferons do not induce the expression of mitoNEET. Therefore, we compared the mitoNEET cellular level with an O_2_‐sensitive [Fe‐S] enzyme, namely, Elp3, whose expression is not induced by interferons. Additionally, Elp3 has the same [4Fe‐4S](Cys)_3_ cluster as interferon‐stimulated hSAND. We tested if the levels of mitoNEET and Elp3 in effector T cells (T_effs_) were affected by hypoxic conditions (Fig. 5D). We chose these T cells because their function and activity in diseases such as cancer is regulated by hypoxia and NO [66, 67]. While the hypoxic condition significantly increased the cellular level of Elp3, it did not substantially affect that of mitoNEET (Fig. 5D). These results suggest that under low O_2_ levels, the [4Fe‐4S] cluster is less degraded, stabilizing the protein and increasing its level, but the mitoNEET [2Fe‐2S] cluster was not affected as expected from our biochemical data showing O_2_ resistance at pH 7.0. Next, we studied if a high O_2_ level (21% O_2_, approximately 254 μm) can protect mitoNEET [2Fe‐2S] cluster against NO desensitization toward H_2_S reduction. To perform this experiment, we first confirm that under our experimental condition, at a mitoNEET and NO‐donor (DEA NONOate) concentration of 16 μm used for NO/O_2_ competition studies (Fig. 5E), NO is released throughout circa 4 h and the reaction of NO with O_2_ is negligible. The mass conservation equation (Data S1) predicts an NO release rate of less than 0.1 μm·s^−1^, at which the reaction of NO with O_2_ is negligible (Fig. S8A,B). This release rate and the availability of NO for approximately 4 h under aerobic conditions were further confirmed using spin‐trap experiments (Fig. S8C–E). We then exposed mitoNEET to NO under normoxic conditions (O_2_ level 254 μm), and after 4 h to remove almost all NO, mitoNEET was treated with H_2_S donor under anaerobic conditions. We found that H_2_S reduced the cluster (Fig. 5E), unlike when mitoNEET was exposed to NO in the presence of very low amount of O_2_ (Fig. 5A). Therefore, high O_2_ levels protect the [2Fe‐2S] cluster against NO reaction and desensitization towards H_2_S reduction. To understand how O_2_ could block NO reaction with the cluster, we performed MD simulations in a mixture of O_2_/NO. We sampled the NO or O_2_ interactions with mitoNEET (Data S1). The average backbone RMSD values of the protein for 50 ns were below 2 Å (Fig. S9A), indicating that the system is in thermal equilibrium and stable. Interaction studies revealed that O_2_ forms hydrogen bonds with residues at the interface between two subunits, namely, Tyr71 and Arg73, and that the NO ability to form hydrogen bonds with these residues is abolished (Fig. 5F). This effect of O_2_ on NO interaction is similar to that we observed for V70W mutation (Fig. 3E). Additionally, the O_2_ survival probability remains near 100% after 20 ns compared to the survival probability of NO, which decreases (Fig. S9B). These predictions suggest that O_2_ blocks NO access to the cluster. Exposure of the reduced V70W or V70C variant to O_2_ led to the formation of oxidized protein (Fig. S9C,D). Therefore, it appears that O_2_ access to the cluster is not solely dependent on the NO access tunnel. Blocking NO access will possibly facilitate the auto‐oxidation of NO by O_2_ before it reaches the cluster.

## Discussion

Previous works with mitoNEET focused on the individual reaction of O_2_, NO or H_2_O_2_ with its [2Fe‐2S] cluster, but the mechanism of the reaction at the molecular level, the competition between NO and O_2_ and the reaction of H_2_S with the cluster were not studied [31, 33, 34, 35, 36, 37, 38, 39, 40, 55]. It was shown that the half‐life of mitoNEET [2Fe‐2S] cluster under aerobic conditions increased exponentially by increasing pH from circa 5 to 6.7 [31, 36, 37]. Decreasing pH to less than 7, a pH value less than the pKa of histidine ligand of the mitoNEET [2Fe‐2S] cluster, makes the cluster labile and unstable [33, 34]. The pH‐dependent liability of the cluster explains why, at low pH, the cluster is not stable in the presence of O_2_. Therefore, it is expected that at pH 7 or above, the mitoNEET cluster is oxygen resistant. Consistently, we found that in phosphate buffer with a pH value of 7.0, the cluster is highly resistant to O_2_ degradation. Similarly, at pH 7, the mitoNEET [2Fe‐2S] cluster is stable in response to H_2_O_2_ exposure and is not degraded [31, 37, 38], consistent with our findings. Adding 500 μm H_2_O_2_ after reducing the cluster with DTT (10 000 μm) was suggested to oxidize the cluster transiently [35]. When we added H_2_O_2_ in the absence of sodium dithionite, we observed that the cluster was stable, and the reduced mitoNEET [2Fe‐2S]^1+^ cluster was oxidized. Finally, under anaerobic conditions, NO oxidizes the cluster of the NEET family of proteins and binds to the cluster to generate iron‐nitrosyl species [38, 39, 40, 55]. Similarly, we observed that NO oxidizes and interacts with the mitoNEET [2Fe‐2S] cluster under anaerobic conditions, generating iron‐nitrosyl species.

Unlike previous reports, here we studied the molecular mechanism of the reaction of O_2_ and gasotransmitters NO and H_2_S with mitoNEET [2Fe‐2S] cluster and investigated how the presence of O_2_ affects these reactions. We first combined MD simulations and mutagenesis studies to elucidate the NO pathway to the cluster. We discovered an explicit NO access tunnel. We showed for the first time that gasotransmitter H_2_S can reduce the mitoNEET [2Fe‐2S] cluster. Based on these findings, we tested whether the O_2_ level affects NO oxidation and H_2_S reduction. When the O_2_ level was very low, exposure of mitoNEET to NO led to the desensitization of the cluster towards H_2_S reduction. However, under normoxic conditions or when pioglitazone was added, NO access to the cluster was blocked and the cluster was not desensitized towards reduction by H_2_S. These data together confirm the presence of an O_2_‐regulated NO access site to the mitoNEET [2Fe‐2S] cluster. These findings also suggest a new biological action of H_2_S. The reaction of gasotransmitter H_2_S with [Fe‐S] proteins, including the NEET family, and its interplay with oxidizing signalling molecules like NO and O_2_ have not been studied previously. H_2_S has multiple functions [68]. It can coordinate with metallocofactors in haemoglobin, reduce disulphide bonds, oxidize radicals like NO and superoxide and form covalent bonds with amino acid residues of proteins [68]. Our data suggest that H_2_S can reduce certain [Fe‐S] clusters.

**Fig. 6:**
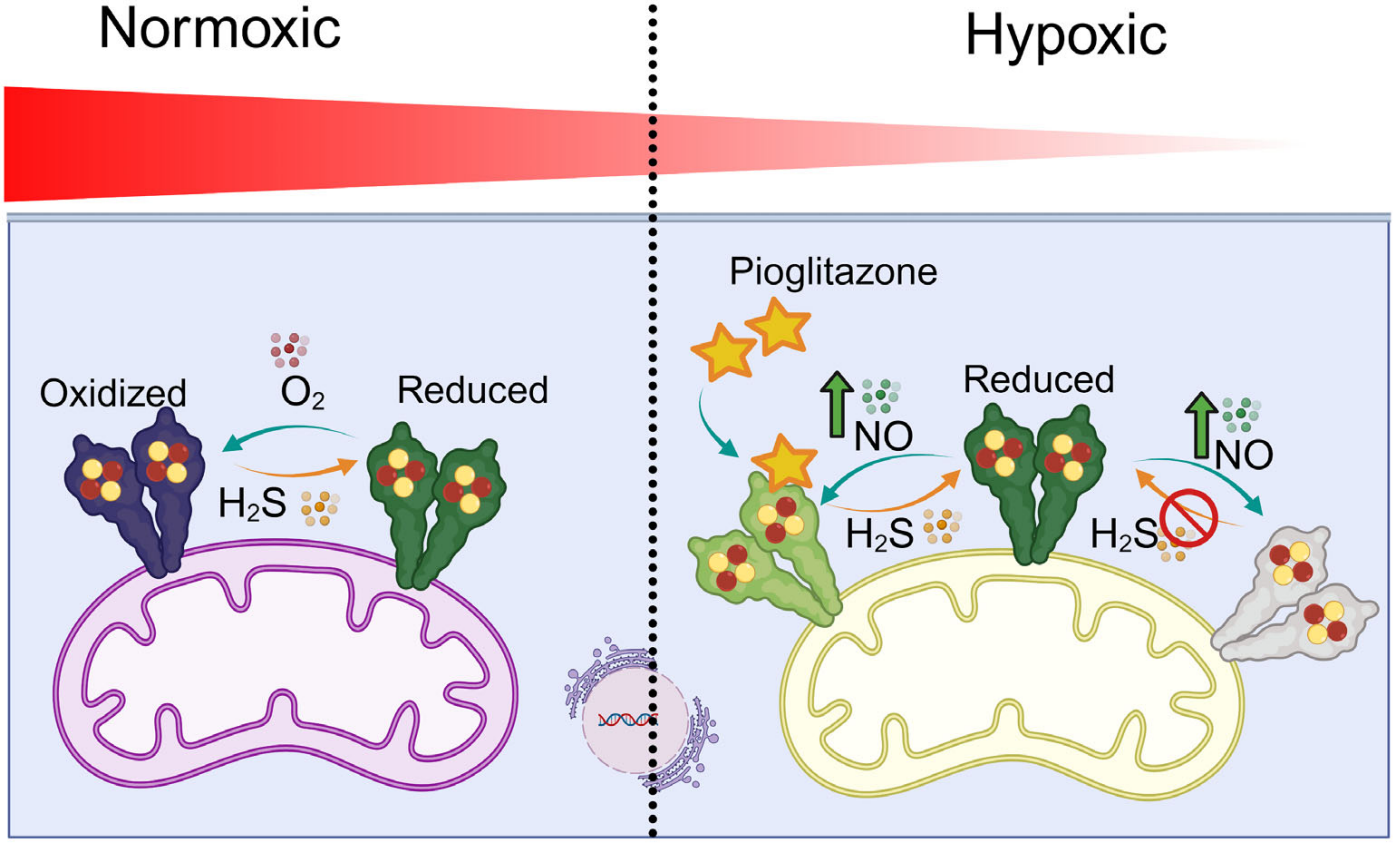
A schematic model describing the molecular function of mitoNEET [2Fe‐2S] cluster in sensing O_2_ level and gasotransmitters. Under normoxic conditions, O_2_ protects the mitoNEET [2Fe‐2S] cluster (left). Under inflammatory and hypoxic conditions (right), when the NO level rises, mitoNEET is oxidized by NO and desensitized to H_2_S reduction (grey). Pioglitazone protects the mitoNEET cluster against NO oxidation and enables H_2_S reduction (light greeen).

Based on our findings, we put forward a molecular mechanism for the interplay among NO, O_2_ and H_2_S reactions with mitoNEET [2Fe‐2S] cluster (Fig. 6). Under hypoxic and inflammatory conditions characterized by an increase and a decrease in the NO and O_2_ levels, respectively, there will be an increase in the reaction of NO with mitoNEET [2Fe‐2S] cluster. The NO reaction desensitizes the oxidized mitoNEET [2Fe‐2S]^2+^ cluster towards reduction by biological reductants like gasotransmitter H_2_S or other biologically relevant reducing agents, for example, anamorsin/Ndor1 complex [69]. As a result, the concentration of the NO‐oxidized and ‐desensitized mitoNEET rises. The presence of pioglitazone restores the balance between the reduced and oxidized mitoNEET. It is known that the redox state of mitoNEET plays an essential role in mitochondrial oxidative capacity and function [23, 24]. The oxidized mitoNEET interacts with VDAC [23], reducing mitochondrial membrane potential. A reduction in mitoNEET expression level enhances mitochondrial respiration [25], and mitoNEET knockout mice show signs of mitochondrial dysfunction and a Parkinson's disease phenotype [27]. While mitoNEET loss leads to mitochondrial dysfunction in B‐cell acute lymphoblastic leukaemia [28], its activity reduces oxidative damage to conserve mitochondrial function [63]. Consequently, based on our findings and model (Fig. 6), we postulate that under hypoxic conditions, an increase in NO‐desensitized mitoNEET contributes to the loss of mitochondrial membrane potential and the formation of dysfunctional mitochondria. By blocking NO access to the cluster, pioglitazone restores the balance between oxidized and reduced mitoNEET, attenuating oxidative damage and restoring mitochondrial function/integrity [29]. Future cell‐based experiments should test the link between our model and the function of mitoNEET in modulating mitochondrial activity.

In conclusion, we discovered an explicit NO access site to the mitoNEET [2Fe‐2S] cluster, suggesting a molecular mechanism of mitoNEET function in gasotransmitter signal transduction. Our data collectively revealed that O_2_ and, similarly, pioglitazone block the NO access and protect the mitoNEET [2Fe‐2S] cluster from NO desensitization. These findings will have broad implications for understanding the pathways *via* which hypoxia affects mitochondrial function and designing new therapeutics to target mitoNEET.

## Author contributions

KHE conceived the project; TNH performed all biochemical measurements; MW‐L carried out computational studies; AC and P‐LH performed EPR measurements; MA and MH carried out experiments with T cells and performed western blot measurements; TNH and KHE designed experiments; all authors contributed to the discussion and analyses of data; TNH and KHE prepared the manuscript draft with contribution from all authors; TNH, SK, P‐LH, MAM, MMR and KHE received funding.

## Supporting information


**Data S1.** Supplementary methods.
**Fig. S1.** Degradation of hSAND cluster by O_2_, H_2_O_2_ and NO.
**Fig. S2.** The O_2_, H_2_O_2_ and NO reaction with mitoNEET [2Fe‐2S] cluster.
**Fig. S3.** Evaluation of protein stability and prediction of gas tunnels and a gas gate in mitoNEET.
**Fig. S4.** V70C mutation does not affect NO interaction with residues near the [2Fe‐2S] cluster.
**Fig. S5.** The mutation of valine 70 to tryptophane (V70W) or to cysteine (V70C) does not affect the [2Fe‐2S] cluster.
**Fig. S6.** H_2_S reaction with hSAND and mitoNEET.
**Fig. S7.** Pioglitazone protects the mitoNEET [2Fe‐2S] cluster from NO reaction.
**Fig. S8.** NO release rate and availability in the presence of O_2_.
**Fig. S9.** MD simulations of mix NO and O_2_ diffusion in mitoNEET.
**Table S1.** MitoNEET residues with C‐α located in a 3.5 Å radius of the [2Fe‐2S] cluster establish contact with H_2_O_2_ and NO molecules (diffusant).
**Table S2.** List of mitoNEET residues that participate in Tunnel 1 and those that are involved in the entry site to the tunnel, according to predictions from the caver web 1.0 server.

## Data Availability

The data, excluding EPR and computational data, that support the findings of this study are openly available on Dryad at https://doi.org/10.5061/dryad.3xsj3txrz, reference number FEBSL‐24‐1135. The EPR and computational data that support the findings of this study are available on request from the corresponding author, as they are not publicly available due to privacy.
