# Supplementary material for: The [2Fe‐2S] cluster of mitochondrial outer membrane protein mitoNEET has an O2 ‐regulated nitric oxide access tunnel
Source: FEBS Lett. 2025 Jan 5;599(7):952–70. doi: 10.1002/1873-3468.15097 (PMC11995679; doi:10.1002/1873-3468.15097)
Supplement: Supplementary file 1 — Data S1. Supplementary methods. Fig. S1. Degradation of hSAND cluster by O2, H2O2 and NO. Fig. S2. The O2, H2O2 and NO reaction with mitoNEET [2Fe‐2S] cluster. Fig. S3. Evaluation of protein stability and prediction of gas tunnels and a gas gate in mitoNEET. Fig. S4. V70C mutation does not affect NO interaction with residues near the [2Fe‐2S] cluster. Fig. S5. The mutation of valine 70 to tryptophane (V70W) or to cysteine (V70C) does not affect the [2Fe‐2S] cluster. Fig. S6. H2S reaction with hSAND and mitoNEET. Fig. S7. Pioglitazone protects the mitoNEET [2Fe‐2S] cluster from NO reaction. Fig. S8. NO release rate and availability in the presence of O2. Fig. S9. MD simulations of mix NO and O2 diffusion in mitoNEET. Table S1. MitoNEET residues with C‐α located in a 3.5 Å radius of the [2Fe‐2S] cluster establish contact with H2O2 and NO molecules (diffusant). Table S2. List of mitoNEET residues that participate in Tunnel 1 and those that are involved in the entry site to the tunnel, according to predictions from the caver web 1.0 server. [file FEB2-599-952-s001.pdf]

The [2Fe-2S] cluster of mitochondrial outer membrane protein mitoNEET has an O_2-regulated nitric oxide access tunnel

Thao Nghi Hoang[1,2], Meritxell Wu-Lu[3], Alberto Collauto[4], Peter-Leon Hagedoorn[5], Madalina Alexandru[1,6], Maike Henschel[1,6], Shahram Kordasti[6], Maria Andrea Mroginski[3], Maxie M. Roessler[4], Kourosh H. Ebrahimi[1*]

[1] Institute of Pharmaceutical Science, King's College London, London, UK

[2] Department of Pharmacy, Da Nang University of Medical Technology and Pharmacy, Da Nang, Viet Nam

[3] Department of Chemistry, Technical University of Berlin, Berlin, Germany

[4] Department of Chemistry and Centre for Pulse EPR spectroscopy (PEPR), Imperial College London, London, UK

[5] Department of Biotechnology, Delft University of Technology, TU Delft, NL

[6] Comprehensive Cancer Center, King's College London, London, UK

Correspondence: Kourosh.ebrahimi@kcl.ac.uk

Supplementary Information Content

**Content**	**Page**
Supplementary Methods	3
Supplementary Figure 1	6
Supplementary Figure 2	7
Supplementary Figure 3	9
Supplementary Figure 4	10
Supplementary Figure 5	11
Supplementary Figure 6	12
Supplementary Figure 7	13
Supplementary Figure 8	14
Supplementary Figure 9	15
Supplementary Table 1	16
Supplementary Table 2	17
References	18

Supplementary Methods

Electron paramagnetic resonance (EPR) measurements. X-band CW-EPR measurements were performed on a Bruker EMX (Figure 2F, SI Figures 2H and 6D) or EMXplus (SI Figure 6B) spectrometer. The EMX spectrometer was equipped with an ER 4122SHQE resonator (Bruker) and a closed-circuit cryostat (Cryogenic Ltd.), and the EMXplus spectrometer was equipped with a helium-flow cryostat operated at 15 K.[1,2] For the EMX spectrometer, the sample temperature was controlled using a Lakeshore 350 temperature controller (Lake Shore Cryotronics, Inc.).

Bruker EMX. The spectra were recorded at a temperature of 40 K using a microwave power of 0.2 mW, a field modulation amplitude of 5 G at 100 kHz, a magnetic field sweep rate of 12 Gs^{-1}, a conversion time of 81.92 ms and a time constant of 20.48 ms. The background signal of the empty cryostat Dewar was recorded using the same acquisition conditions and subtracted from the spectra. The magnetic field was calibrated using DPPH as a standard (g = 2.0036).[3]

Bruker EMXplus. EPR conditions: Microwave frequency, 9.405 GHz; Microwave power, 2 mW; modulation frequency, 100 kHz; modulation amplitude, 10 G; temperature, 15 K. The magnetic field was calibrated using the Bruker BDPA (1,3-bis(diphenylene)-2-phenylallyl radical) standard with a g-value of 2.00254 ± 0.00003.

For the H_2S-reduced and NO-oxidised samples power saturation measurements (conducted on the Bruker EMX spectrometer) were performed at a temperature of 40 K to characterise the spin relaxation behaviour; the same acquisition parameters described above were used. The results were analysed using the following equation:[4]

$I_{pp} = I_0 \cdot \frac{\sqrt{P}}{(1 + P/P_{½})^{b/2}}$	(equation S1)

in which P is the microwave power, the parameter b describes the homogeneous character of the EPR line ($b = 1$ for an inhomogeneously broadened line and $b = 3$ for a homogeneously broadened line) and $P_{½}$ is the microwave power at which the saturation factor $s = (1 + \gamma_e^2 B_1^2 T_1 T_2)^{-1}$ is equal to ½.

For the H_2S-reduced sample, the peak-to-peak amplitude of the g_y feature was used for the analysis, whereas for the NO-oxidised sample, the peak-to-peak amplitude of the whole spectrum was considered. The results, shown in SI Figure 2H, highlight a considerably slower spin relaxation for the iron-dinitrosyl complex compared to the [2Fe-2S] cluster.

X-band pulse EPR measurements were performed on a Bruker Elexsys E580 spectrometer equipped with an ER 4118X-MS-2 split-ring resonator (Bruker), a closed-circuit cryostat (Cryogenic Ltd.), a 1 kW travelling-wave tube amplifier (Applied Systems Engineering, Inc., model 117X), a SpinJet arbitrary waveform generator (Bruker) and a SpecJet-III digitiser (Bruker). The sample temperature was controlled using a Lakeshore 350 temperature controller (Lake Shore Cryotronics, Inc.). To enhance the detection sensitivity, the microwave resonator was installed on a modified probehead[5] (Amplify My Probe Ltd.) containing a 36 dB cryogenic low-noise amplifier (Low Noise Factory AB, model LNF-LNC4_16C) and a 6 dB directional coupler (Pasternack Enterprises, Inc.).

HYSCORE spectra were recorded at a temperature of 10 K at the field position corresponding to the maximum of the g_y feature in the echo-detected EPR spectrum using the 4-pulse sequence.[6]

$$(\pi/2)_1 - \tau - (\pi/2)_2 - t_1 - (\pi) - t_2 - (\pi/2)_3 - \tau - echo$$

A shot repetition time of 5.1 ms was used.

The length of all pulses was set to 12 ns; an inter-pulse delay τ of 134 ns was used, corresponding to placing the second blind-spot at the ^{1}H Larmor frequency to suppress the diagonal peak from matrix protons. The time intervals t_1 and t_2 were incremented independently from a starting value of 80 ns in 20-ns steps; this latter value corresponds to a Nyquist frequency of 25 MHz. 256 points were recorded along each time dimension, corresponding to a maximum time of 5.18 µs on each axis. The stimulated echo was integrated over a 10 ns gate.

An 8-step phase cycle[7] was used:

$(\pi/2)_1$	$(\pi/2)_2$	π	$(\pi/2)_3$	$Acq.$
+x	+x	+x	+x	+1
+x	+x	+x	-x	-1
+x	+x	-x	+x	+1
+x	+x	-x	-x	-1
+x	-x	+x	+x	-1
+x	-x	+x	-x	+1
+x	-x	-x	+x	-1
+x	-x	-x	-x	+1

The datasets were processed using home-written scripts based on EasySpin 5.2.36[8] and running on MATLAB R2021a (The MathWorks, Inc.). Briefly, the spectra, acquired using quadrature detection, were background-corrected along the t_1 and t_2 dimensions using a 3rd order polynomial, apodised along both time dimensions using a Hamming window, zero-filled to a final size of 1024×1024 and 2D Fourier transformed. Lastly, the absolute value of the 2D Fourier transform was symmetrised along the diagonal ($S_{symm} = (SS^T)^{1/2}$)[9] and displayed as a contour plot.

Molecular Dynamics (MD) Simulations. The simulation systems were constructed using the crystal structure of the mitoNEET protein (PDB code 2QH7)[10] as initial geometry. The settings were starting point residue B:500; probe radius 0.5; shell radius 3.0; shell depth 4.0; frame weighting coefficient 1.0; and frame clustering threshold 1.0. The protein's protonation pattern was determined using Karlsberg2+,[11] assuming pH 7.0. Consequently, all titratable residues were in their standard protonation state, and all histidine residues were single-protonated at Nε. The protein was immersed into a cubic box of TIP3P model water[4] of dimension 150 Å x 150 Å x 150 Å and further neutralized by adding 150 mM NaCl. This solvated model system served as the basis for generating four distinct simulation models: 1) mitoNEET protein with 200 molecules of H_2O_2, 2) mitoNEET protein with 200 molecules of NO, 3) mitoNEET protein with 200 molecules of H_2S, and 4) mitoNEET protein with 100 molecules of NO and 100 molecules of O_2. All diffusing molecules were randomly added to the water box using Packmol.[12]

Protein atoms and diffusing small molecules were modelled using the CHARMM36 force field [13]while force field parameters for the [2Fe-2S] cluster was were obtained from published studies made by Pesce *et al*.[14] All MD simulations were performed under the same condition with NAMD version 2.14.[15,16] The time step of 2 fs was enabled by the Shake algorithm[17] that constrains the bond lengths of hydrogen atoms. Van der Waals potential was truncated at 12 Å and the particle-Mesh Ewald (PME)[18] method was used to calculate long-distance electrostatic interactions. Temperature control was achieved with Langevin dynamics at 300K and flexible cell.[19]

The four structural models were energy minimized for 75000 steps with the ABNR algorithm and thermally equilibrated at 300 K with harmonic constraints applied to all heavy atoms and the metal centre for 500 ps. The constrains on all heavy atoms except for those on the [2Fe-2S] cluster were lifted before production. Each MD simulation was run for 300 ns in an NPT ensemble at 300K and 1 bar pressure and repeated 3 times. Only the last 50 ns of each MD run were used for statistical analysis. The CAVER Web 1.0 server[20] was employed to analyze accessible tunnels within the protein, utilizing the following parameters: the starting point was the [2Fe-2S] cluster, shell depth (4 Å), shell radius (3 Å), cluster threshold (3.5 Å) desired radius (5 Å), and maximum distance (3 Å) were maintained constant for all calculations.

Visualization of structures and images was done using Visual Molecular Dynamics (VMD)[21] and PyMOL for visualizing the tunnels along with custom codes developed in Python3,8 utilizing packages such as Numpy1.20.3 and MDAnalysis2.0-0 8.[22]

Pioglitazone docking. For docking studies mitoNEET structure (PDB code 3REE) was used. Molecular docking was performed using the open-source software PyRx, as explained previously.[23,24]

Analysis of [Fe-S] protein level in effector T (T_{effs}) cells. Human peripheral blood mononuclear cells (PBMCs) from healthy donors were used (Ethic Approval 23-EE-0005). Cells were thawed in RPMI medium (Gibco) supplemented with 10% FBS (Sigma Aldrich). A CD4+CD25+ regulatory T cell isolation kit (Miltenyi Biotec) was used to isolate T_{effs} according to the manufacturer's protocol. The isolate T_{effs} were cultured using RPMI medium (10% FBS) in an incubator, 37 °C and 5% CO_2 under normoxic (20% O2) or hypoxic conditions (1% O_2), the latter using a hypoxia incubator chamber (Stemcell Technologies) as described previously.[25] An inflammatory condition was induced by the addition of inflammatory cytokines IFN-γ (R&D Systems, 285-IF) and IL-1β (R&D Systems, 201-LB) (10 ng/ml). After 96 hours, cells were collected by centrifugation at 300xg for 5 minutes at 4 C. Cells were washed with PBS, and the cell pellet was stored at -80 C freezer for subsequent analysis using western blot. Cells were lysed using RIPA buffer containing protease and phosphatase inhibitors (ThermoFisher). For western blot analysis of cell lyase, 12, 14, or 20% SDS gels were prepared using the SureCast Gel Handcast System (Invitrogen), according to the manufacturer's protocol. Alternatively, Bolt 4-12% Bis-Tris Plus gels (Invitrogen) were used. After running SDS gel, the proteins were transferred onto a PVDF membrane (ThermoFisher) using the Power Blotter system (ThermoFisher) and subsequently, blocking and antibody incubation were performed using the iBind Flex system (ThermoFisher) according to the manufacturer protocol. All primary antibodies were purchased from Proteintech. The primary antibodies were Rabbit anti-GAPDH (1:1000), Rabbit anti-human Elp3 (1:1000) or Rabbit anti-human CISD1 (1:500 or 1:1000). The secondary antibody was Goat anti-rabbit HRP-conjugated (Cell Signalling) (1:1000). Clarity Western ECL Substrate (Bio-Rad) was used for visualisation and imaging was performed using an iBright imaging system (ThermoFisher). The exposure time was 8 minutes. The protein ladder was PageRuler Plus Prestained Protein ladder (ThermoScientific) or MagicMark™ XP Western Protein Standard.

Supplementary Figures

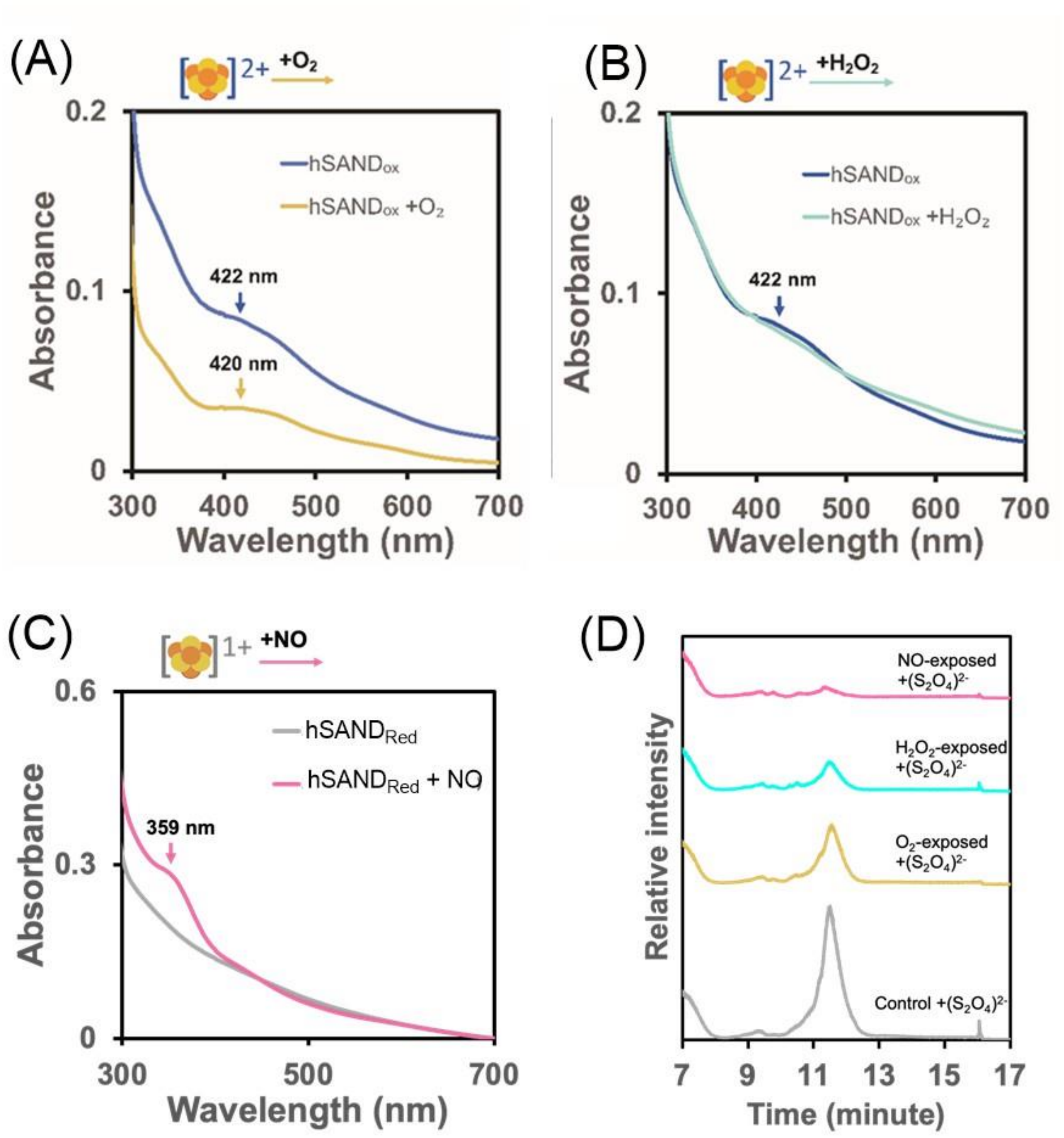

Supplementary Fig. 1. Degradation of hSAND cluster by O_2, H_2O_2, and NO. (A) Oxidized hSAND ($hSAND_{Ox}$) was exposed to molecular oxygen (4 hours) or **(B)** hydrogen peroxide (4 hours under anaerobic conditions). The concentrations of hSAND and H_2O_2 were 8 µM and 16 µM, respectively. **(C)** The reaction of reduced hSAND ($hSAND_{Red}$) (30 µM) with NO (NO-donor concentration of 30 µM) (4 hours under anaerobic conditions). To reduce hSAND, sodium dithionite was used (30 min). **(D)** hSAND exposed to O_2, H_2O_2, or NO is catalytically inactive. The enzyme was exposed to O_2, H_2O_2, or NO (4 hours under anaerobic conditions) and then used to prepare reactions as explained in online methods. The concentrations of hSAND, NO-donor and H_2O_2 were 40 µM, 40 µM and 47 µM, respectively. Those of sodium dithionite $(S_2O_4)^{2-}$, SAM and CTP were 3.5 mM, 14 mM and 3.5 mM, respectively. The graph shows the extracted ion chromatogram of ddhCTP $([M-H]^{-1}$ m/z of 464.0) generated by hSAND. All experiments were repeated three times to confirm reproducibility. Buffer was (a-c) phosphate 50 mM, 100 mM NaCl, or (d) MOPS 50 mM, 100 mM NaCl, pH 7.0. All experiments were performed at room temperature (~22 °C) under anaerobic conditions (O_2 < 5 ppm).

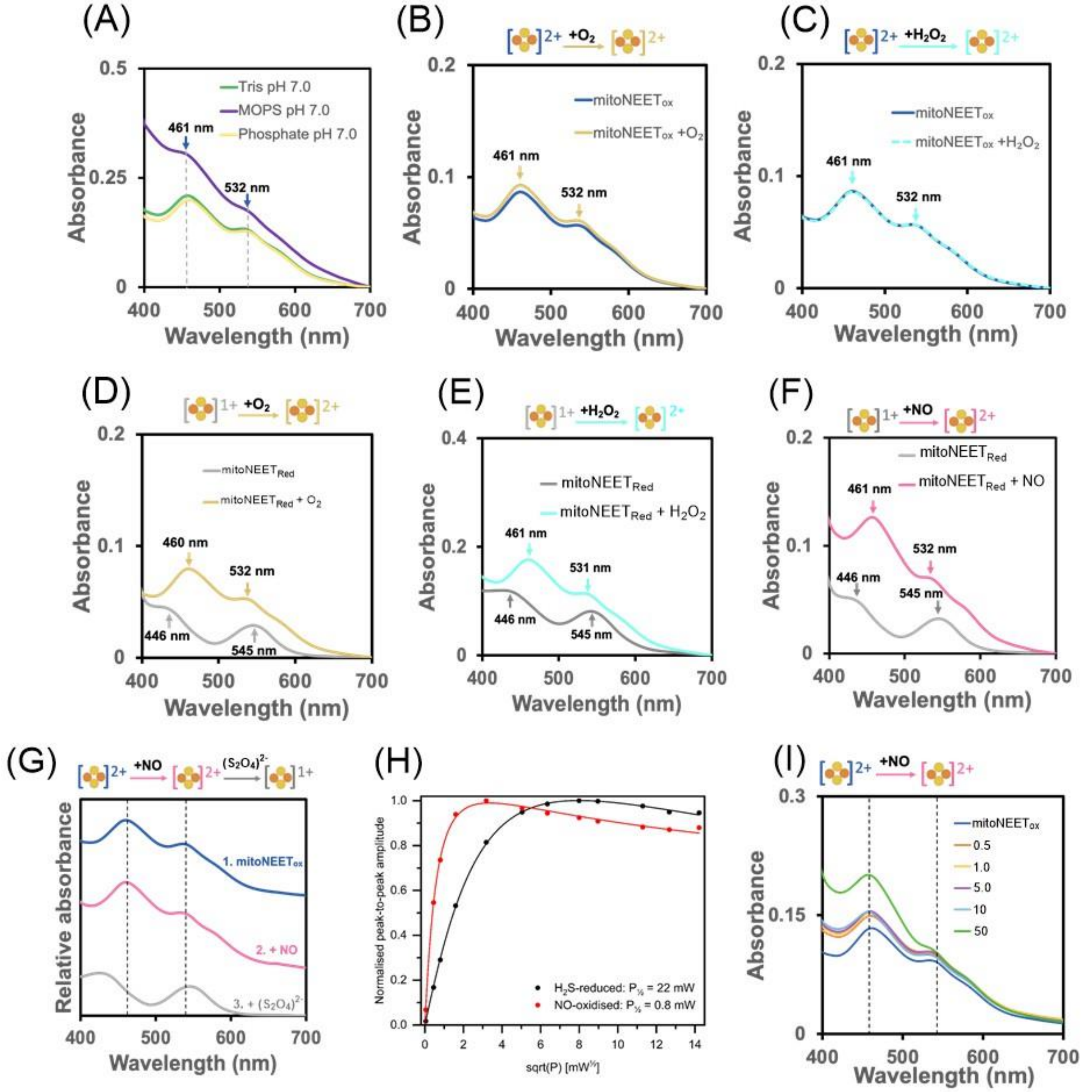

Supplementary Fig. 2. The O_2, H_2O_2, and NO reaction with mitoNEET [2Fe-2S] cluster. (A) Effect of different buffers on the mitoNEET [2Fe-2S] cluster. The mitoNEET concentration was 30 µM. **(B)** The oxidised mitoNEET $[2Fe\text{-}2S]^{2+}$ cluster (mitoNEET$_{Ox}$) does not react with O_2 or **(C)** H_2O_2 (4 hours incubation) **(D)** The reduced mitoNEET $[2Fe\text{-}2S]^{1+}$ cluster (mitoNEET$_{Red}$) is oxidized by O_2 (4 hours) or **(E)** H_2O_2 (4 hours). To reduce mitoNEET, sodium dithionite was added anaerobically to mitoNEET (30 min incubation). **(B-D)** The final concentrations of mitoNEET, H_2O_2, and sodium dithionite ($(S_2O_4)^{2-}$) were 10 µM, 11 µM, and 10 µM, respectively. (e) The final concentrations of mitoNEET, H_2O_2, and sodium dithionite ($(S_2O_4)^{2-}$) were 25 µM, 22 µM, and 25 µM, respectively. Sodium dithionite was removed before the addition of H_2O_2. **(F)** The reaction of ($(S_2O_4)^{2-}$)-reduced $[2Fe\text{-}2S]^{1+}$ cluster with NO (4 hours incubation). The mitoNEET concentration was (10 µM). Concentrations of sodium dithionite and NO-donor were 10 µM and 1 mM, respectively. **(G)** Sodium dithionite $(S_2O_4)^{2-}$ can reduce mitoNEET $[2Fe\text{-}2S]^{2+}$ after its exposure to NO. The concentration of mitoNEET was 19 µM. The ratio of dithionite and NO-donor to mitoNEET was one. **(H)** Analysis of the power saturation data for the X-band CW-EPR spectra of the H_2S-reduced (black dots) and NO-oxidised (red dots) [2Fe-2S] mitoNEET cluster measured at 40 K. The solid lines correspond to a fitting of the data performed using equation S1; the relevant parameters are $P_{½}$ = 21.7 mW, b = 1.45 for the H_2S-

reduced cluster and $P_{½}$ = 0.81 mW, b = 1.21 for the NO-oxidised cluster. The concentration of mitoNEET, H_2S-donor and NO-donor were 200 µM, 430 µM and 215 µM. **(I)** NO titration of oxidized mitoNEET (4 hours incubation time). The final concentrations of mitoNEET was 16 µM. The stock concentration of NO-donor was 1.12 mM, and an aliquot of the stock was added to reach NO-donor to mitoNEET ratio of 0.5, 1, 5, 10, or 50. The final volume was always 700 µL. All experiments were repeated three times to confirm reproducibility. Buffer was phosphate 50 mM, 100 mM NaCl, pH 7. All experiments were performed at room temperature (~22 °C).

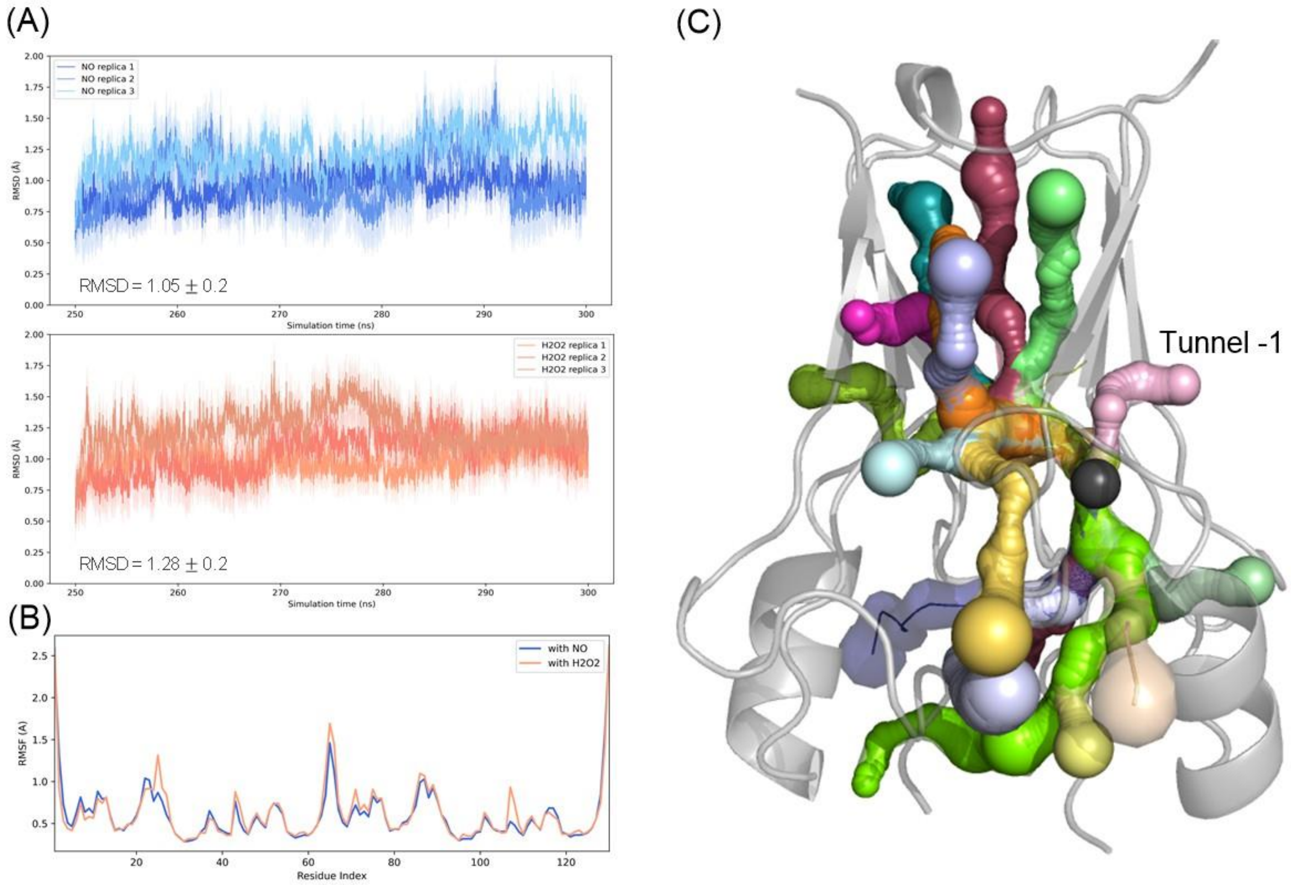

Supplementary Fig. 3. Evaluation of protein stability and prediction of gas tunnels and a gas gate in mitoNEET. (A) Evolution of Root-mean-square-deviation (RMSD) values of all protein heavy atoms relative to initial mitoNEET structure (PDB code 2QH7) over the last 50 ns of the three MD repeats. *Top:* NO diffusion simulations, *Bottom:* H_2O_2 diffusion simulations. **(B)** Evaluation of root-mean-square fluctuations (RMSF) of mitoNEET residues during H_2O_2- (orange trace) and NO- (blue trace) diffusion MD simulations taking all repeats into account. RMSF unit is Ångstrøm. **(C)** 19 access tunnels of NO to the [2Fe-2S] cluster of mitoNEET resulting from CAVER Web 1.0 analysis. Tunnel-1 (in pink) provides direct access to the cluster.

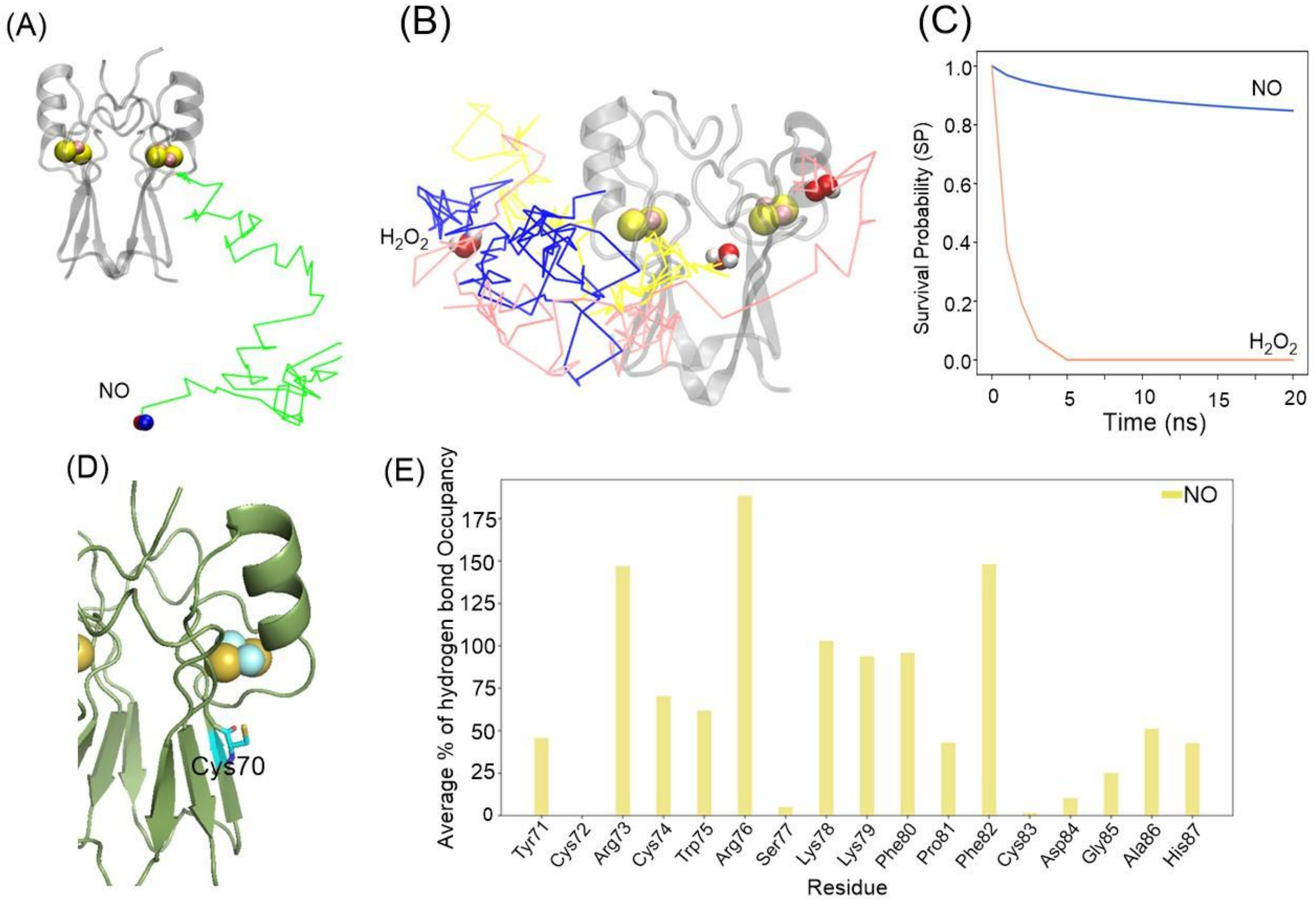

Supplementary Fig. 4. V70C mutation does not affect NO interaction with residues near the [2Fe-2S] cluster. A) The MD simulations of wild-type mitoNEET predict the trajectory of NO (Green) and **(B)** H_2O_2 (blue, salmon, or yellow) movement towards the [2Fe-2S] cluster. **(C)** The survival probability (SP) of NO (blue) or H_2O_2 (salmon) in the wild-type mitoNEET. **(D)** Energy-optimized structure of mitoNEET-V70C variant. The results predict that NO access was not affected by the V70C mutation. **(E)** Percentage of hydrogen bonds formed between NO molecule and individual residues of mitoNEET-V70C variant during the last 50 ns of the NO-diffusion simulation repeats. Hydrogen bonds were identified by using the cut-off distance of 3.5 Å between hydrogen bond donor/acceptor atoms and a bond angle cut-off of 120°. All calculations were repeated three times to confirm reproducibility.

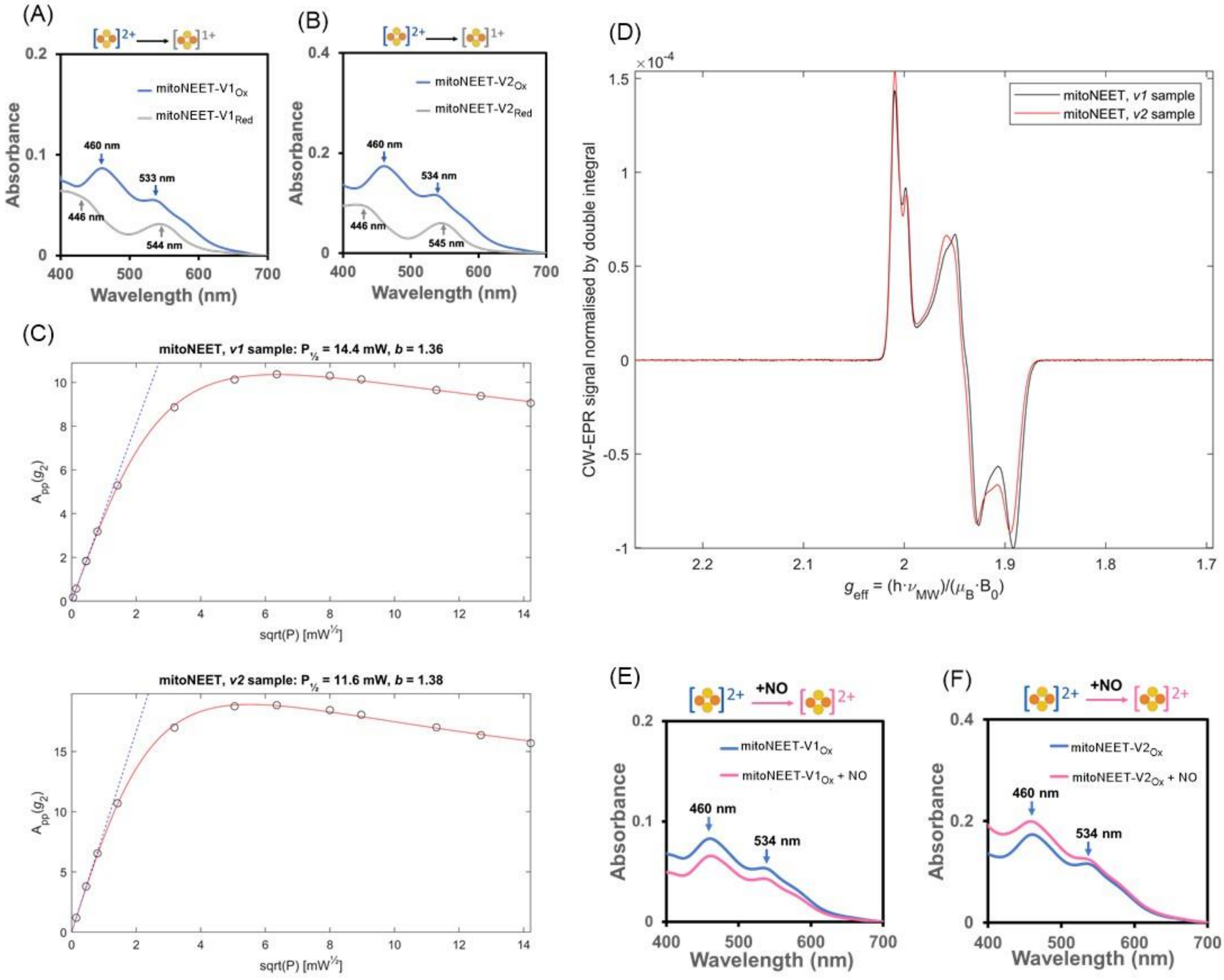

Supplementary Fig. 5. The mutation of valine 70 to tryptophane (V70W) or to cysteine (V70C) does not affect the [2Fe-2S] cluster. (A) The UV-visible absorbance spectrum of the fully oxidized and reduced (30 min sodium dithionite) cluster in V70W (V1) and **(B)** V70C (V2) variants. The concentrations of V70W and V70C variants were 14 µM and 25 µM, respectively, and that of dithionite was two times the protein. **(C)** Analysis of the power saturation data for the X-band CW-EPR spectra of the dithionite reduced (black dots) [2Fe-2S] of V70W (160 µM) and V70C (260 µM) variants measured at 40 K. The ratio of sodium dithionite $(S_2O_4)^{2-}$ to protein was 10:1. **(D)** X-band CW-EPR spectra at 40 K of sodium dithionite-reduced mitoNEET-V70W (v1 sample, 160 µM) [2Fe-2S] cluster (black trace) as compared to dithionite-reduced mitoNEET-V70C (v2 sample, 260 µM) [2Fe-2S] cluster (red trace). The ratio of sodium dithionite $(S_2O_4)^{2-}$ to protein was 10:1. **(E)** The fully oxidized mitoNEET-V70W (V1) (14 µM) or **(F)** oxidized mitoNEET-V70C (V2) (25 µM) variant was exposed to NO (4 hours incubation), and UV-visible absorbance spectra were recorded before (blue) and after (pink) exposure. As Isolated proteins were exposed to oxygen to ensure they were fully oxidized. The proteins were then incubated in the anaerobic glovebox and exposed to NO under anaerobic conditions. (a-b & e-f) Experiments were repeated at least two times and performed at room temperature (~22 °C) and under anaerobic conditions (O_2 < 5 ppm). Buffer was phosphate 50 mM, 100 mM NaCl, pH 7.

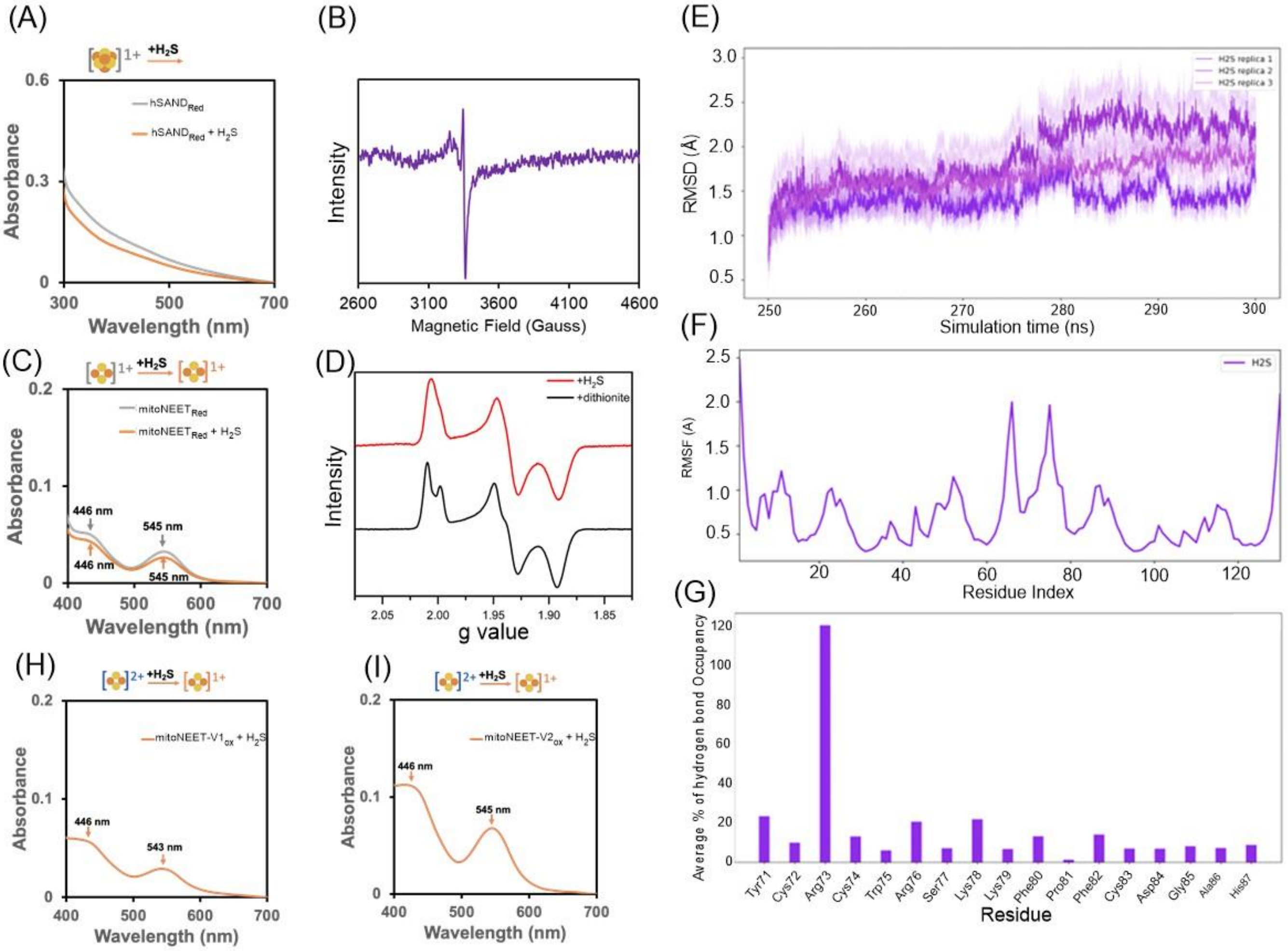

Supplementary Fig. 6. H_2S reaction with hSAND and mitoNEET. (A) hSAND was reduced by the addition of sodium dithionite. Subsequently, the H_2S-donor solution was added under anaerobic conditions. After adding sodium dithionite, the sample was incubated under anaerobic conditions for circa 10 min, and subsequently, H_2S-donor was added (4 hours incubation). The final concentrations of hSAND and H_2S-donor were 31 µM and 28 µM, respectively. Sodium dithionite ($(S_2O_4)^{2-}$) 31 µM (final concentration) was used to reduce hSAND. **(B)** EPR spectroscopy confirmed that H_2S does not reduce the $[4Fe\text{-}4S]^{2+}$ cluster of SAND. The final concentration of hSAND was 112.5 µM, and that of H_2S-donor was 37.8 mM. **(C)** Exposure to H_2S (4 hours) does not change the spectrum of reduced mitoNEET ($mitoNEET_{Red}$). MitoNEET was first reduced by adding sodium dithionite (30 min) and subsequently exposed to the H_2S (4 hours). The spectra were recorded before and after the addition of H_2S-donor. Concentrations of H_2S-donor and mitoNEET were 1 mM and 10 µM, respectively. **(D)** X-band CW-EPR spectra at 40 K of sodium dithionite-reduced mitoNEET [2Fe-2S] cluster (black trace) as compared to H_2S-reduced mitoNEET [2Fe-2S] cluster (red trace). Concentrations of mitoNEET, sodium dithionite and H_2S-donor were 200 µM, 215 µM and 430 µM. **(E)** Evolution of Root-mean-square-deviation (RMSD) values of all protein heavy atoms relative to initial mitoNEET structure (PDB code 2QH7) over the last 50 ns of the three MD repeats. **(F)** RMSF (root-mean-square fluctuations in Ångstrøm) of mitoNEET residues during H_2S diffusion MD simulations taking all repeats into account. **(G)** Percentage of hydrogen bonds formed between the H_2S molecule and individual residues of the mitoNEET during the last 50 ns of the three H_2S-diffusion simulation repeats. Hydrogen bonds were identified by using the cut-off distance of 3.5 Å between hydrogen bond donor/acceptor atoms and a bond angle cut-off of 120°. **(H)** UV-visible absorbance spectrum of H_2S-reduced (4 hours) mitoNEET-V70W and **(I)** mitoNEET-V70C. The concentrations of V70W and V70C variants were 14µM and 25 µM, respectively, and that of H_2S-donor was equal to the concentration of protein. All experiments and calculations were repeated three times to confirm reproducibility. Buffer was (a, c-i) phosphate 50 mM, 100 mM NaCl, pH 7.0 or (b) MOPS 50 mM, 100 mM NaCl, pH 7.0. (a, c, h, i) Experiments were performed at room temperature (~22 °C) and under anaerobic conditions (O_2 < 5 ppm).

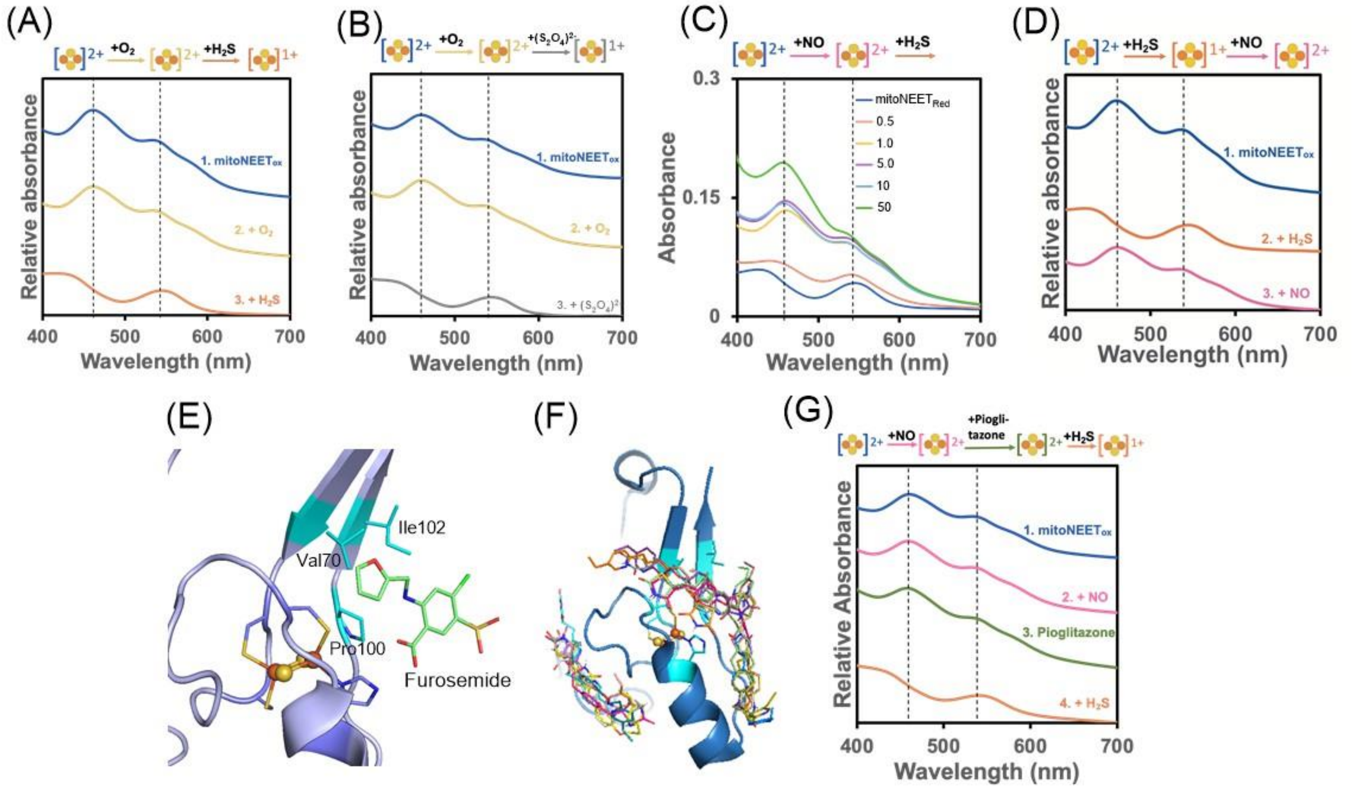

Supplementary Fig. 7. Pioglitazone protects the mitoNEET [2Fe-2S] cluster from NO reaction. (A) Adding H_2S (4 hours incubation under anaerobic conditions) to O_2-exposed mitoNEET (16 µM) reduces the cluster. The ratio of H_2S donor to mitoNEET was one. **(B)** Sodium dithionite reduces O_2-exposed mitoNEET. The final concentrations of mitoNEET and sodium dithionite ($(S_2O_4)^{2-}$) were 8 µM and 10 µM, respectively. **(C)** H_2S reduction of NO-titrated mitoNEET. MitoNEET was exposed (overnight anaerobically) to different amounts of NO and then exposed to H_2S (4 hours). The final concentrations of mitoNEET and H_2S-donor were 16 µM. The values next to each trace show the NO to mitoNEET ratio. **(D)** NO oxidises H_2S-reduced mitoNEET. The protein was exposed (overnight anaerobically) to H_2S and then to NO (4 hours anaerobically). The concentration of mitoNEET was 16 µM. The ratio of NO-donor or H_2S-donor to mitoNEET was one. **(E-F)** Thiazolidinedione ligands bind to the residues, forming the NO entry site to the shallow tunnel-1. **(E)** Crystal structure of soluble mitoNEET with sulfonamide ligand (PDB code: 6DE9). The ligand blocks NO entry to tunnel-1. **(F)** The result of pioglitazone docking into the structure of mitoNEET monomer (PDB Code: 3REE). The results predict the binding of pioglitazone at different positions, including on the top of the surface, forming the NO entry to tunnel-1. **(G)** Incubation of NO-oxidized mitoNEET with pioglitazone re-sensitised the cluster towards H_2S reduction. MitoNEET was exposed to NO (4 hours) and subsequently overnight to pioglitazone, all under anaerobic conditions. It was then exposed to H_2S (four hours under anaerobic conditions) The concentration of mitoNEET was 19 µM. The ratio of NO-donor, H_2S-donor, or pioglitazone to mitoNEET was one. (a-d, g) Buffer was phosphate 100 mM, 300 mM NaCl, pH 7.0. Experiments were performed at room temperature (~22 °C) under anaerobic conditions. All experiments and calculations were repeated three times to confirm reproducibility.

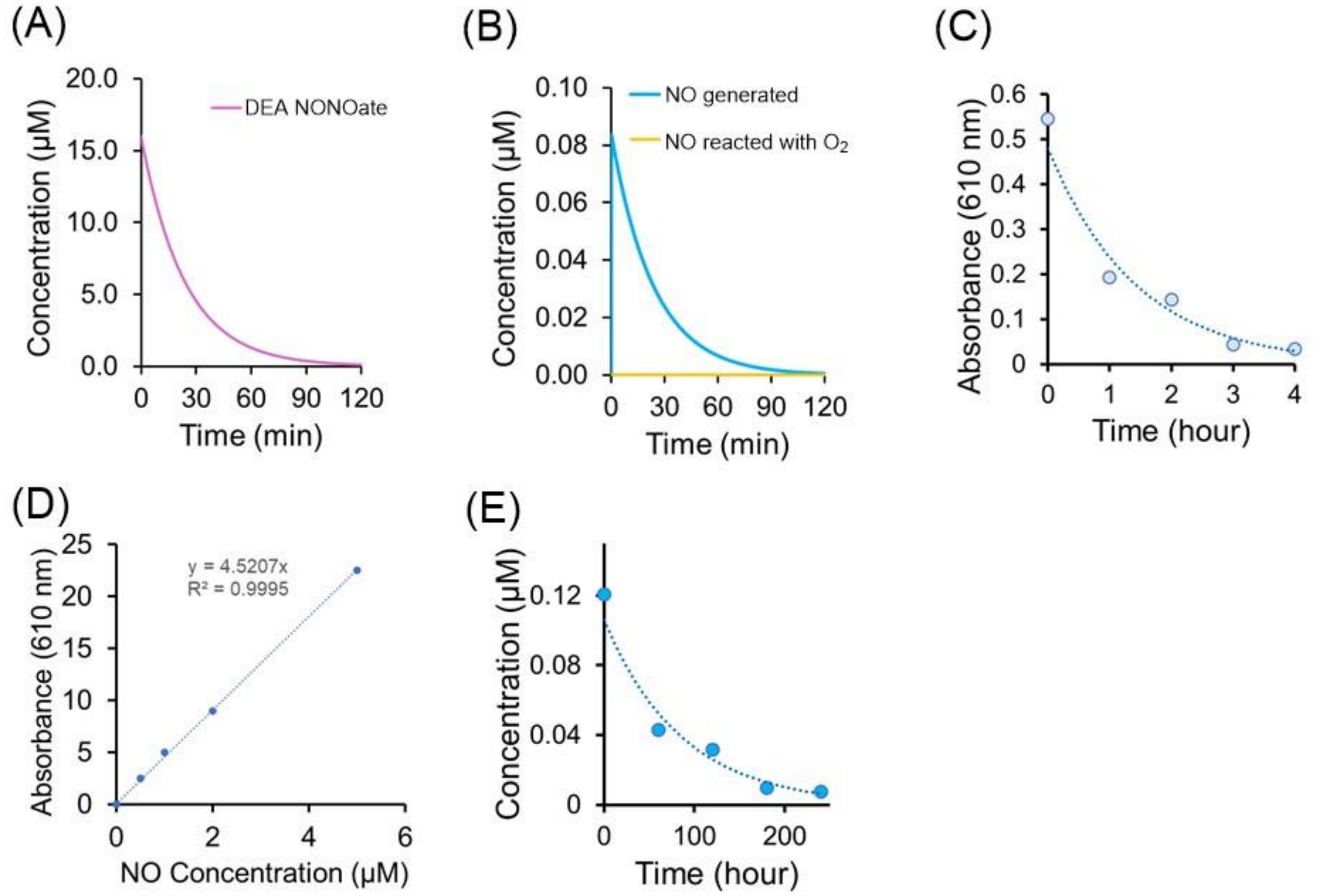

Supplementary Fig. 8. NO release rate and availability in the presence of O_2. (A) The calculated amount of DEA NONOate as a function of time (pH 7.0) using equation 1 (Methods). **(B)** The amount of NO released in the presence of O_2 using equation 2 (Methods) (blue) and the amount of NO reacted with O_2 (Orange). (a-b) The initial DEA NONOate concentration was16 µM (used for NO and O_2 competition studies). The calculation shows that NO is released gradually. **(C)** Kinetics of NO release under aerobic conditions as measured using TMPDA (the concentrations of TMPDA and NO-donor were 21 µM. Samples were prepared as explained in the methods and absorbance was measured at 610 nm**. (D)** Standard curve for conversion of absorbance of TMPDA after trapping NO as a function of NO concentration. **(E)** The concentration of NO generated by NONOate under aerobic concentration is plotted as a function of time. The concentrations were obtained using the standard curve (D) and the values in (C). The observed kinetic of NO generated under an oxygenic environment correlates very well with the predicted kinetics in (B). This confirms that at low NONOate concentration and under our experimental condition, the reaction of NO with O2 is negligible. Buffer was phosphate 50 mM, 100 mM NaCl, pH 7. Samples were prepared at room temperature (~22 °C) under aerobic conditions.

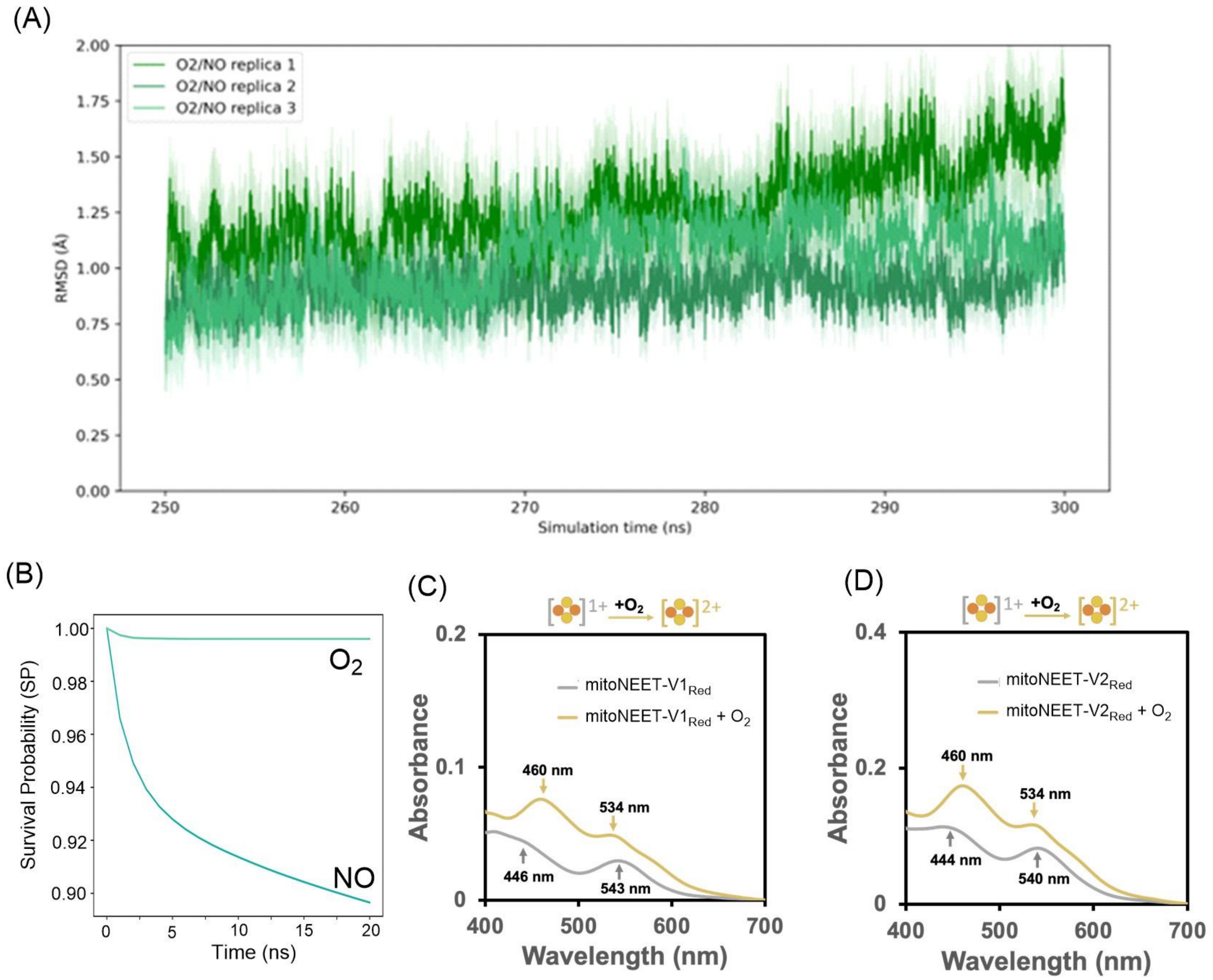

Supplementary Fig. 9. MD simulations of mix NO and O_2 diffusion in mitoNEET. (A) Evolution of Root-mean-square-deviation (RMSD) values of all protein heavy atoms relative to initial mitoNEET structure (PDB code 2QH7) over the last 50 ns of the three MD repeats; **(B)** The survival probability of O_2 and NO is calculated in a mixture of O_2/NO. **(B)** UV-visible absorbance of reduced V70W and then oxidized by O_2 and **(D)** reduced V70C and then oxidized by O_2 (under aerobic conditions for four hours). The concentrations of V70W and V70C variants were 14 and 25 µM, respectively. The ratio of sodium dithionite ($(S_2O_4)^{2-}$) to mitoNEET was one. Buffer was phosphate 50 mM, 100 mM NaCl, pH 7. (c-d) Samples were prepared at room temperature (~22 °C).

Supplementary Tables

Supplementary Table 1. MitoNEET residues with C-α located in a 3.5 Å radius of the [2Fe-2S] cluster that establish a contact with H_2O_2 and NO molecules (diffusant). A contact is established when the minimum distance between small-molecule and protein heavy atoms is below 3.5 Å.

	**Involved residues**
**H_2O_2**	Arg76, Ser77, Lys78, Lys79, Phe80, Pro81, Phe82, Cys83, Asp84, Gly85, Hse87
**Pure NO**	Tyr71, Cys72, Arg73, Arg76, Ser77, Lys78, Lys79, Phe80, Pro81, Phe82, Cys83, Asp84, Gly85, Ala86, Hse87

Supplementary Table 2. List of mitoNEET residues that participate in Tunnel 1 and those that are involved in the entry site to the tunnel, according to predictions from the CAVER Web 1.0 server

	**Residues**	**Bottle neck**
**Tunnel 1**	Val57, His58, Ala69, Val70, Tyr71, Cys72, Arg73, Cys74, Ser77, Pro81, Phe82, Cys83, Asp84, Gly85, His87, Gly99, Pro100, Leu101, Ile102.	Val70, Tyr71, Cys72, Arg73, Pro100, Leu101, Phe82, Gly99

References

1. Salmeen, I. & Palmer, G. Electron Paramagnetic Resonance of Beef-Heart Ferricytochrome c. *J. Chem. Phys.* **48**, 2049–2052 (1968).

2. Lundin, A. & Aasa, R. A simple device to maintain temperatures in the range 4.2–100 K for EPR measurements. *J. Magn. Reson.* **8**, 70–73 (1972).

3. Krzystek, J., Sienkiewicz, A., Pardi, L. & Brunel, L. C. DPPH as a Standard for High-Field EPR. *J. Magn. Reson.* **125**, 207–211 (1997).

4. van der Est, A., Goldfarb, D. & Stoll, S. Continuous-wave EPR. *EPR Spectrosc. Fundam. Methods* 8–9 (2018).

5. Šimėnas, M. *et al.* A sensitivity leap for X-band EPR using a probehead with a cryogenic preamplifier. *J. Magn. Reson.* **322**, 106876 (2021).

6. Höfer, P., Grupp, A., Nebenführ, H. & Mehring, M. Hyperfine sublevel correlation (hyscore) spectroscopy: a 2D ESR investigation of the squaric acid radical. *Chem. Phys. Lett.* **132**, 279–282 (1986).

7. Stoll, S. & Kasumaj, B. Phase Cycling in Electron Spin Echo Envelope Modulation. *Appl. Magn. Reson.* **35**, 15–32 (2008).

8. Stoll, S. & Schweiger, A. EasySpin, a comprehensive software package for spectral simulation and analysis in EPR. *J. Magn. Reson.* **178**, 42–55 (2006).

9. Fábregas Ibáñez, L. *et al.* Non-uniform HYSCORE: Measurement, processing and analysis with Hyscorean. *J. Magn. Reson.* **307**, 106576 (2019).

10. Paddock, M. L. *et al.* MitoNEET is a uniquely folded 2Fe–2S outer mitochondrial membrane protein stabilized by pioglitazone. *Proc. Natl. Acad. Sci.* **104**, 14342–14347 (2007).

11. Meyer, T. & Knapp, E.-W. Karlsberg+: A tool to predict pKa values and study proton transfer pathways in proteins using electrostatic energy calculations. *BBA-Bioenergetics* e112 (2014).

12. Jorgensen, W. L., Chandrasekhar, J. & Madura, J. D. Comparison of simple potential functions for simulating liquid water. *J. Chem. Phys.* **79**, 926–935 (1983).

13. Huang, J. & MacKerell Jr, A. D. CHARMM36 all-atom additive protein force field: Validation based on comparison to NMR data. *J. Comput. Chem.* **34**, 2135–2145 (2013).

14. Pesce, L. *et al.* Molecular dynamics simulations of the [2Fe–2S] cluster-binding domain of NEET proteins reveal key molecular determinants that induce their cluster transfer/release. *J. Phys. Chem. B* **121**, 10648–10656 (2017).

15. Nelson, M. T. *et al.* NAMD: a parallel, object-oriented molecular dynamics program. *Int. J. Supercomput. Appl. High Perform. Comput.* **10**, 251–268 (1996).

16. Phillips, J. C. *et al.* Scalable Molecular Dynamics with NAMD. *J. Comput. Chem.* **26**, 1781–1802 (2005).

17. Ryckaert, J.-P., Ciccotti, G. & Berendsen, H. J. C. Numerical integration of the cartesian equations of motion of a system with constraints: molecular dynamics of n-alkanes. *J. Comput. Phys.* **23**, 327–341 (1977).

18. Darden, T., York, D. & Pedersen, L. Particle mesh Ewald: An N· log (N) method for Ewald sums in large systems. *J. Chem. Phys.* **98**, 10089–10092 (1993).

19. Martyna, G. J., Tobias, D. J. & Klein, M. L. Constant pressure molecular dynamics algorithms. *J. Chem. Phys.* **101**, 4177–4189 (1994).

20. Stourac, J. *et al.* Caver Web 1.0: identification of tunnels and channels in proteins and analysis of ligand transport. *Nucleic Acids Res.* **47**, W414–W422 (2019).

21. Humphrey, W., Dalke, A. & Schulten, K. VMD: Visual molecular dynamics. *J. Mol. Graph.* **14**, 33–38 (1996).

22. Gowers, R. J. *et al.* MDAnalysis: a Python package for the rapid analysis of molecular dynamics simulations. in *Proceedings of the 15th python in science conference* vol. 98 105 (SciPy Austin, TX, 2016).

23. Dragelj, J., Mroginski, M. A. & Ebrahimi, K. H. Hidden in plain sight: Natural products of commensal microbiota as an environmental selection pressure for the rise of new variants of SARS-CoV-2. *ChemBioChem* **22**, 2946–2950 (2021).

24. Da, A., Wu-Lu, M., Dragelj, J., Mroginski, M. A. & Ebrahimi, K. H. Multi-structural molecular docking (MOD) combined with molecular dynamics reveal the structural requirements of designing broad-spectrum inhibitors of SARS-CoV-2 entry to host cells. *Sci. Rep.* **13**, 16387 (2023).

25. Kosti, P., Larios-Martinez, K. I., Maher, J. & Arnold, J. N. Generation of hypoxia-sensing chimeric antigen receptor T cells. *STAR Protoc.* **2**, 100723 (2021).
